# Marker-assisted introgression of three dominant blast resistance genes into an aromatic rice cultivar *Mushk Budji*

**DOI:** 10.1038/s41598-018-22246-4

**Published:** 2018-03-06

**Authors:** Gazala Hassan Khan, Asif Bashir Shikari, Rakesh Vaishnavi, Sofi Najeeb, Bilal A. Padder, Zahoor A. Bhat, Ghulam A. Parray, Mohammad Ashraf Bhat, Ram Kumar, Nagendra K. Singh

**Affiliations:** 1 Division of Plant Biotechnology, Sher-e-Kashmir University of Agricultural Sciences and Technology of Kashmir, Shalimar Campus, Srinagar, J & K India; 2 DARS, Old Airport Road, Sher-e-Kashmir University of Agricultural Sciences and Technology of Kashmir, Budgam, J & K India; 3 Mountain Research Centre for Field Crops, Sher-e-Kashmir University of Agricultural Sciences and Technology of Kashmir, Khudwani, J & K India; 4 Division of Plant Pathology, Sher-e-Kashmir University of Agricultural Sciences and Technology of Kashmir, Shalimar Campus, Srinagar, J & K India; 5 Division of Genetics and Plant Breeding, Sher-e-Kashmir University of Agricultural Sciences and Technology of Kashmir, FOA, Wadura, Sopore, Baramullah, J & K India; 6 ICAR- National Research Centre on Plant Biotechnology, Lal Bahadur Shastri Building, Pusa Campus, New Delhi, India

## Abstract

Modern high yielding rice varieties have replaced most of the traditional cultivars in recent past. *Mushk Budji*, is one such short grained landrace known for its aroma and exquisite quality, however, is highly susceptible to blast disease that has led to considerable decline in its area. *Mushk Budji* was crossed to a triple-gene donor line, DHMAS 70Q 164-1b and followed through marker-assisted foreground and background selection in first and second backcross generations that helped to incorporate blast resistance genes *Pi54, Pi1* and *Pita*. Marker-assisted background selection was carried out using 78 SSR and STS markers that helped to reduce linkage drag around the genes *Pi54, Pi1* and *Pita* to 2.74, 4.60 and 2.03 Mb, respectively. The three-gene lines in BC_2_F_2:3_ were genotyped using 50 K SNP chip and revealed more than 92% genome similarity to the RP. 2-D gel assay detected differentially expressing 171 protein spots among a set of backcross derived lines, of which 38 spots showing match score of 4 helped us to calculate the proteome recovery. MALDI-TOF analysis helped to detect four significant proteins that were linked to quality and disease resistance. The improved lines expressed resistance to blast under artificial and natural field conditions.

## Introduction

In India, rice forms staple food for more than 70% of population where around 4,000 varieties and hybrids are grown to gratify varied consumer preferences^1^. India, a country encompassing Centre of origin of rice, is endowed with enormous genetic diversity and is home to at least 50,000 landraces of rice^2^. Overtly, North-west Himalayan region of the country has in recent past, witnessed considerable genetic erosion and decline in area under local scented and non-scented landraces, as a result of preference for high yielding varieties and susceptibility to diseases like blast, brown spot, sheath blight, etc^3^. Within this broad region, although, more than 100 rice landraces have been documented from Kashmir valley^4^ (1500 to 1800 m amsl), only few japonica types are grown presently, which include *Mushk Budji, Kamad, Nun-beoul* and *Zag*. The short grained *Mushk Budji* happens to be the most popular and enjoys prodigious commercial demand due to pleasant aroma and taste of its cooked rice. Grown over 10,000 ha area, its cultivation has shrunken to specific pockets of Kashmir due to heavy incidence of rice blast. A program on *in-situ* conservation of *Mushk Budji* and its revival was concluded successfully^5^, however, an area expansion could not be achieved as expected due to scourge of rice blast disease that causes around 70% yield loss^6^. The plant protection measures have been less economical, unsafe and practically difficult to be undertaken in hilly terrains where *Mushk Budji* is grown. Dwindling genetic resources and constant disease pressure have put a wrath on scented rices as globally they account for only about 2.2% and 1.8% of total cultivars grown in temperate and tropical rice ecologies, respectively^7^. Therefore, the present study was aimed at incorporation of major genes in this particular valuable landrace to make it durably resistant against blast pathogen, *Magnaporthe oryzae*.

Recently, marker-based strategies to incorporate genetic resistance have proven to be successful in development of disease resistant varieties^8–10^. Most of such efforts relied on pyramiding multiple R-genes through marker-assisted backcross breeding (MAB). Single gene resistance often breaks down easily after few years of cultivation due to dynamic nature of blast fungus and its capability to mutate in response to vertical resistance that ultimately renders the host R-genes ineffective shortly after the release of variety. Since, the probability of a given race to overcome a combination of genes in a gene pyramid happens to be extremely low^11^, therefore, pyramiding of multiple blast resistance genes into susceptible backgrounds for achieving durable blast resistance was thought of to be an effective solution. Almost 347 QTLs^9^ and 102^12^ genes have been reported for blast resistance of which 27 (*Pita*, *Pib, Pb1*, *Pizt*, *Pid2*, *Pii*, *Pikm*, *Pit*, *Pid3*, *Pid3-A4*, *Pish*, *Pik*, *Pikp*, *Pia*, *PiCO39*, *Pi1*, *Pi2*, *Pi5*, *Pi9*, *Pi21*, *Pi25*, *Pi33*, *Pi36*, *Pi37*, *P50, Pi54*, and *Pi65(t)*) have been cloned. Presently, we had chosen to pyramid a combination of *Pi54, Pi1* and *Pita* in our recipient parent (RP) with an expectation to provide high degree of resistance to prevalent isolates. The gene *Pi54* was earlier reported to show resistance to diverse isolates of *M. oryzae* across North-west Himalayan region^9,13^. *Pi54* belongs to NBS-LRR class of genes and directs the synthesis of β-1, 3-glucan in response to pathogen challenge. The IC-17 race of the pathogen is predominant in *M. oryzae* population of Kashmir and is avirulent towards *Pita*^6^. Besides, *Pita* has been reported to confer resistance against most of the races worldwide^14^. *Pita* is more of constitutive in its expression and the coded protein bears alanine at 918 position in all the varieties and wild accessions carrying the gene^14,15^. *Pi1* is comparably a weak gene but is effective in Kashmir and is even better performing in combination with other genes.

The marker-assisted selection (MAS) approach has recently been instrumental in transfer of major genes/QTLs in mega varieties of rice^16–19^. MAS entails the methods^20^ like simultaneous or step wise transfer of genes in RP from multiple donors, each carrying a single gene. To serve our purpose our choice was to use a three-gene non-scented donor DHMAS 70Q 164-1b that simplified the scheme of backcrossing. The present study helped us to develop a blast resistant version of *Mushk Budji* for release in farmer’s fields which in long term, may help to relapse the diminishing area under this valuable rice cultivar.

## Results

### Marker-assisted backcrossing

Marker Assisted Backcross Breeding (MABB) strategy was employed to transfer major blast resistance genes *Pi54, Pi1* and *Pita* from a non-aromatic three-gene donor DHMAS70Q 164-1b which was crossed as a male to popular aromatic landrace *Mushk Budji*. Hybridity in F_1_s was confirmed and a single F_1_ was backcrossed to RP *Mushk Budji* to yield 17 BC_1_F_1_ plants. Foreground selection was exercised on BC_1_F_1_ plants to identify heterozygous individuals by using gene based InDel marker *Pi54* MAS for the gene *Pi54*, linked SSR marker RM224 for *Pi1* and gene based coupling-repulsion marker pair YL155/87 and YL 155/83 for the gene *Pita*. Selected BC_1_F_1_ plants were advanced to BC_2_F_1_ and subsequently followed through selfing generations to identify plants carrying homozygosity at target loci. (Supplementary Figs S1 and S2).

Polymorphism survey was carried out between RP *Mushk Budji* and three gene donor line DHMAS 70Q 164-1b using 278 genome wide markers of which 96 markers were found to be polymorphic between parents. The polymorphic markers uniformly distributed across the genome were used to carry out background analysis. A total of 55 and 47 markers were screened for carrier chromosomes 11 and 12, where 11 and 14 markers were found to be polymorphic between the parents, respectively. A starting three-gene BC_1_F_1_ plant, SKUA-485-27 (*Pi54*+*Pi1*+*Pita*) was screened using polymorphic markers and recorded recipient genome recovery (RPG) at 60.87 per cent of polymorphic loci. The plant revealed heterozygous segments at markers RM190 (linked to *Wx* allele), PKN7 and PKN10 which are linked to rice grain quality.

The BC_2_F_1_ plants were analyzed for recombination breakpoint between *Pi54* and *Pi1* on chromosome 11L. A single plant namely, SKUA-485-27-6 carrying *Pi54*+*Pi1* showed recovery at RM254. The three gene plant viz., SKUA-485-27-7 had RP allele at RM26746 (16.8 cM) at proximal end of the *Pi54 *locus while, SKUA-485-27-4 and SKUA-485-27-18 were found to be heterozygous at this marker. Other plants carrying *Pi54*+*Pi1* (SKUA-485-27-2, SKUA-485-27-6, SKUA-485-27-1) and *Pi54*+*Pita* (SKUA-485-27-15) also carried recombination break point at marker RM26746. Further, the individuals carrying three and two genes involving *Pita* were tested for RM144 adjacent to *Pi1* locus. SKUA-485-27-4 and SKUA-485-27-7 carried recombination break point at marker RM144 and SKUA-485-27-1 and SKUA-485-27-2 had RP allele at this locus. Besides, the individuals carrying three and two genes involving *Pita* or *Pita* alone were tested for RM5939 and RM27933 adjacent to *Pita* locus. The plants SKUA-485-27-7 revealed RP allele at RM5939 while rest of the plants showed heterozygous alleles. Further, all the plants were heterozygous at RM27933 except SKUA-485-27-1.

Nine plants showing recovery at markers flanking target genes were subjected to background analysis using 78 genome wide markers distributed across the genome. Of these, eight polymorphic markers were located on each of the chromosomes 1, 6, 11 and 12. Chromosome 4 carried seven polymorphic markers. Six markers each were used for background selection on chromosomes 2, 5, 7 and 10, besides five markers each for chromosomes 3, 8 and 9. The RPG recovery ranged from 63.04% for SKUA-485-27-9 (*Pi54*+*Pi1*) to the maximum value of 79.16% for SKUA-485-27-1 (*Pita*) and SKUA-485-27-15 (*Pi54*+*Pita*). The three gene positive plants SKUA-485-27-7 and SKUA-485-27-4 recorded an RPG of 70.83 and 77.08 per cent, respectively. SKUA-485-27-4 amplified heterozygous alleles at PKN7, RM598 and RM7048. SKUA-485-27-7 was heterozygous at RM190, BADH2, PKN7, RM16301, RM598, RM30, RM7048 and RM160. SKUA-485-27-18 showed heterozygosity at RM170, RM190, RM204 RM314, RM598 and RM160. The plants SKUA-485-27-2, SKUA-485-27-6, SKUA-485-27-9 (all carrying *Pi54*+*Pi1*) and SKUA-485-27-15 (carrying *Pi54*+*Pita*) showed RPG of 68.75, 68.75, 63.04 and 79.16% respectively.

The triple heterozygotes (SKUA-485-27-7 and SKUA-485-27-4), two- and single gene BC_2_F_1_ plants were selfed through BC_2_F_2_ to BC_2_F_2:3_ in order to recover homozygous, two- and three-gene pyramided lines (PLs) and those with individual genes. Also, selected three-gene BC_1_F_1_ plant SKUA-485-27 was advanced to BC_1_F_3:4_. The plants in selfing generations were screened using markers that were heterozygous in previous generation in order to select for RP allele. The scheme and number of plants screened and selected at each generation is given in (Supplementary Fig. S1). The evaluation was carried in early backcross generations on individual plant basis for agronomic traits, cooking quality and target blast resistance loci using foreground markers and is detailed in Supplementary Tables S1–S8.

### Recurrent parent genome recovery in the R-gene pyramided lines

**Figure 1:**
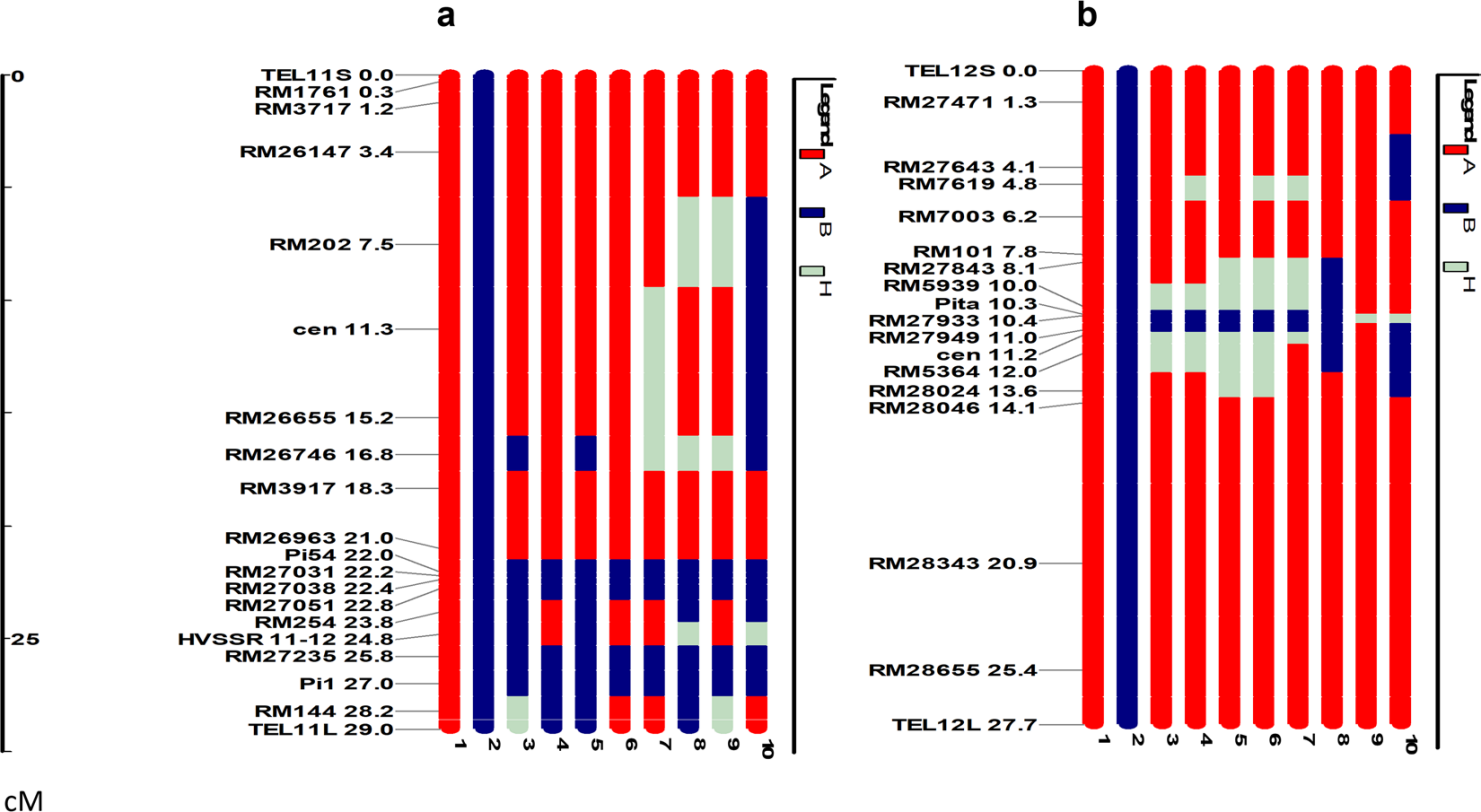
Graphical genotype showing RPG recovery of pyramided lines for carrier chromosome 11 and 12. (**a**) Chromosome 11; (**b**) Chromosome 12; Bar number 1-10: Mushk Budji; DHMAS 70Q 164-1b; SKUA-485-27-4-38-4; SKUA-485-27-4-40-6; SKUA-485-27-3-7-5; SKUA-485-27-77-6-2; SKUA-485-27-20-6-4; SKUA-485-27-86-10-4; SKUA-485-27-47-4-1; SKUA-485-27-13-1-3; A: Recurrent parent allele; B: Donor allele; H: Heterozygous allele; cen: Centromere; TEL: Telomere; S: Short arm; L: Long arm; Distance is given in mega base.

The PLs in BC_1_F_3:4_ and BC_2_F_2:3_ after validation of foreground markers, were analyzed for RPG recovery using genome wide SSR and genic STS markers. The PLs SKUA-485-27-4-38-4, SKUA-485-27-4-40-6, SKUA-485-27-3-7-5, SKUA-485-27-47-4-1 and SKUA-485-27-77-6-2 recorded background genome recovery of 91.03, 83.33, 89.74, 87.18 and 82.69%, respectively (Table 1). The linkage drag around *Pi54* gene was reduced to 2.74 Mb between markers RM254 and RM26963. The, gene *Pi1* was incorporated in SKUA-485-27-47-4-1 within a donor genome segment of 4.6 Mb between the markers RM254 and RM144. Similarly, the *Pita* gene was introgressed within a genome segment of 2.03 Mb between markers RM5939 and RM5364 in the BC_2_F_2:3_ three-gene PLs (Fig. 1; Supplementary Figs S3–5).

**Table 1:** Recipient Parent Genome recovery of pyramided lines.

**S. No**.	**Plant ID**	**Gene**	**RPG recovery (%) (SSR**/**InDels)**	RPG recovery (%) (SNPs)*	**RPG similarity (%) (SNPs)**
1	*Mushk Budji*	—	—	—	100.00
2	DHMAS 70Q 164-1b	*Pi54*+*Pi1*+*Pita*	—	—	56.20
3	SKUA-485-27-4-38-4	*Pi54*+*Pi1*+*Pita*	91.03	87.34	92.60
4	SKUA-485-27-4-40-6	*Pi54*+*Pi1*+*Pita*	83.33	80.71	90.75
5	SKUA-485-27-3-7-5	*Pi54*+*Pi1*+*Pita*	89.74	85.83	91.48
6	SKUA-485-27-13-1-3	*Pi54*+*Pi1*	80.77	69.29	83.07
7	SKUA-485-27-20-6-4	*Pi54*+*Pi1*+*Pita*	85.26	75.97	87.68
8	SKUA-485-27-47-4-1	*Pi54*+*Pi1*	87.18	83.18	89.90
9	SKUA-485-27-77-6-2	*Pi54*+*Pi1*+*Pita*	82.69	71.33	86.66
10	SKUA-485-27-86-10-4	*Pi54*+*Pi1*+*Pita*	80.13	70.25	85.71
11	SKUA-485-27-50-5-5	*Pi54*+*Pi1*+*Pita*	81.41	—	—

*The 50k SNP data for recurrent parent, donor and derived PLs is provided in Supplementary Table S15.

**Figure 2:**
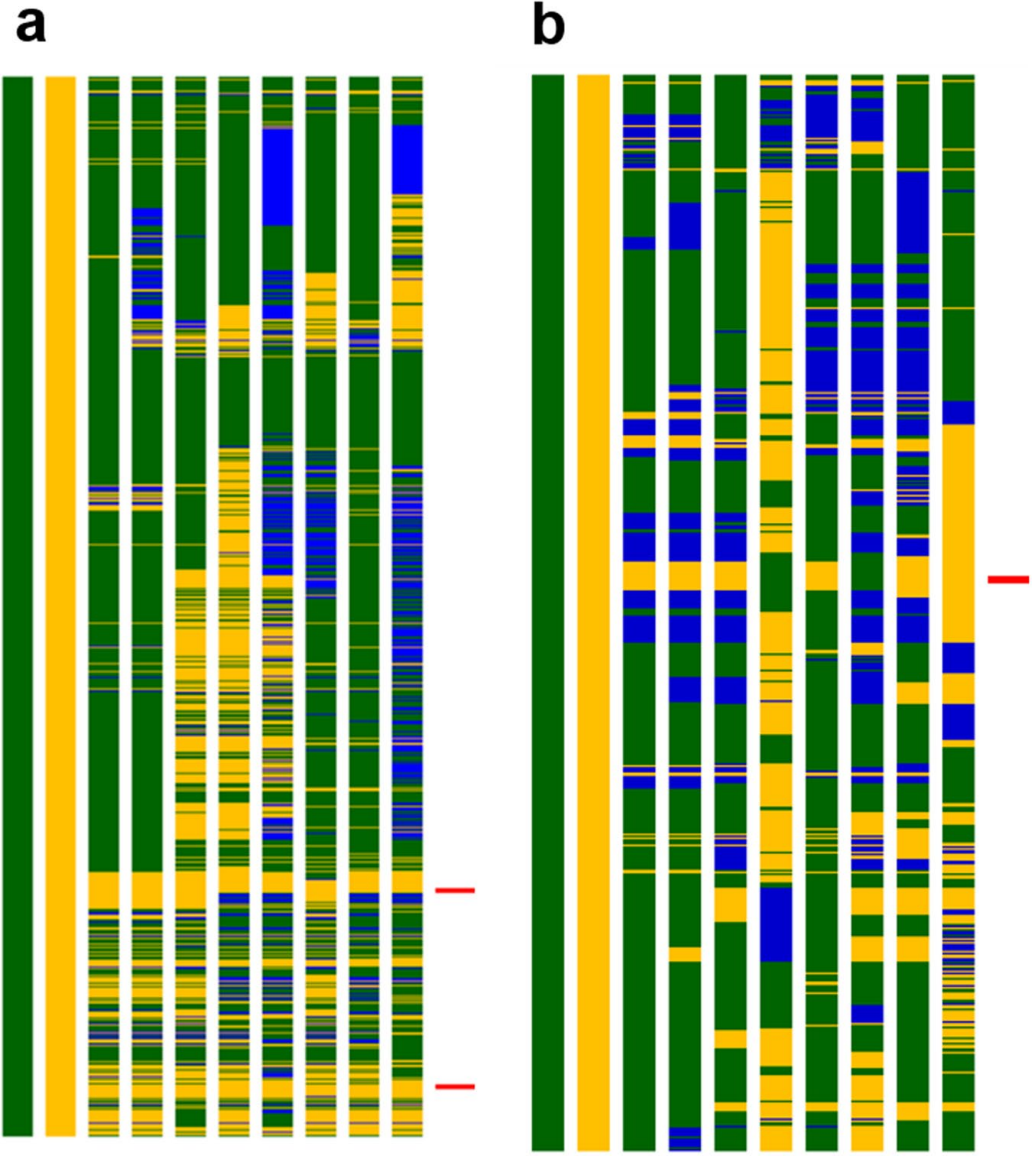
RPG recovery of pyramided lines based on ‘OsSNPnks’ 50 K Axiom^®^ 2.0 SNP array for carrier chromosome 11 and 12. (**a**) Chromosome 11; (**b**) Chromosome 12; Bar number 1-10: Mushk Budji; DHMAS 70Q 164-1b; SKUA-485-27-4-38-4; SKUA-485-27-4-40-6; SKUA-485-27-3-7-5;SKUA-485-27-13-1-3; SKUA-485-27-20-6-4; SKUA-485-27-47-4-1; SKUA-485-27-77-6-2; SKUA-485-27-86-10-4; Yellow: Recurrent parent allele; Green: Donor allele; Blue: Heterozygous allele; Red bars mark the position of genes *Pi54* (above) and *Pi1* (below) on chromosome 11 and *Pita* on chromosome 12.

Eight backcross derived lines and the two parental lines were also genotyped using 50 K SNP chip ‘OsSNPnks’ which carried 50,051 SNPs spread across the twelve rice chromosomes with an average marker density of 131 SNPs per Mb region. The highest number of 10,016 markers was located on rice chromosome 1, followed by Chromosome 3 with 7,044 markers. Both ‘genome similarity’ as well as ‘genome recovery’ with respect to RP was worked out for gene pyramids (Table 1). Three-gene (*Pi54*+*Pi1*+*Pita*) homozygous PLs, namely SKUA-485-27-4-38-4 and SKUA-485-27-3-7-5 showed 92.60% and 91.48% genome similarity, respectively with the RP *Mushk Budji*. For carrier chromosome 11, the similarity percentage of 91.54 and 89.21 were recorded for SKUA-485-27-4-38-4 and SKUA-485-27-4-40-6, respectively. In two and three gene PLs, *Pi54* gene introgression was delineated to a narrow genomic region of about 2 Mb with recombination break points at 19.53 Mb in proximal end and 21.67 Mb at distal end. The *Pi1*gene was located at 24 Mb region with recombination break points at 22.79 in proximal and at 28.21 Mb at telomeric end. For *Pita* gene on carrier chromosome 12, similarity percentage of 91.71 and 89.79 was recorded for SKUA-485-27-4-38-4 and SKUA-485-27-3-7-5, respectively. The average genome similarity of 91.61% was revealed for BC_2_F_2:3_ as compared to 85.80% for the BC_1_F_3:4_ lines. For three-gene PLs SKUA-485-27-4-40-6, SKUA-485-27-3-7-5 and SKUA-485-27-86-10-4 carrying *Pi1*+*Pita*, the linkage drag was reduced to 0.5 Mb on either side of the gene. SKUA-485-27-20-6-4 showed the minimum linkage drag for the *Pita* gene. SKUA-485-27-4-38-4 (BC_2_F_2:3_) carrying *Pi54*+*Pi1*+*Pita* and SKUA-485-27-47-4-1 (BC_1_F_3:4_) carrying *Pi54*+*Pi1* recorded RPG recovery of 87.34 and 83.18%, respectively (Fig. 2), Table 1; Supplementary Fig. S6).

### Recurrent parent proteome recovery in the blast R-gene pyramided lines

**Figure 3:**
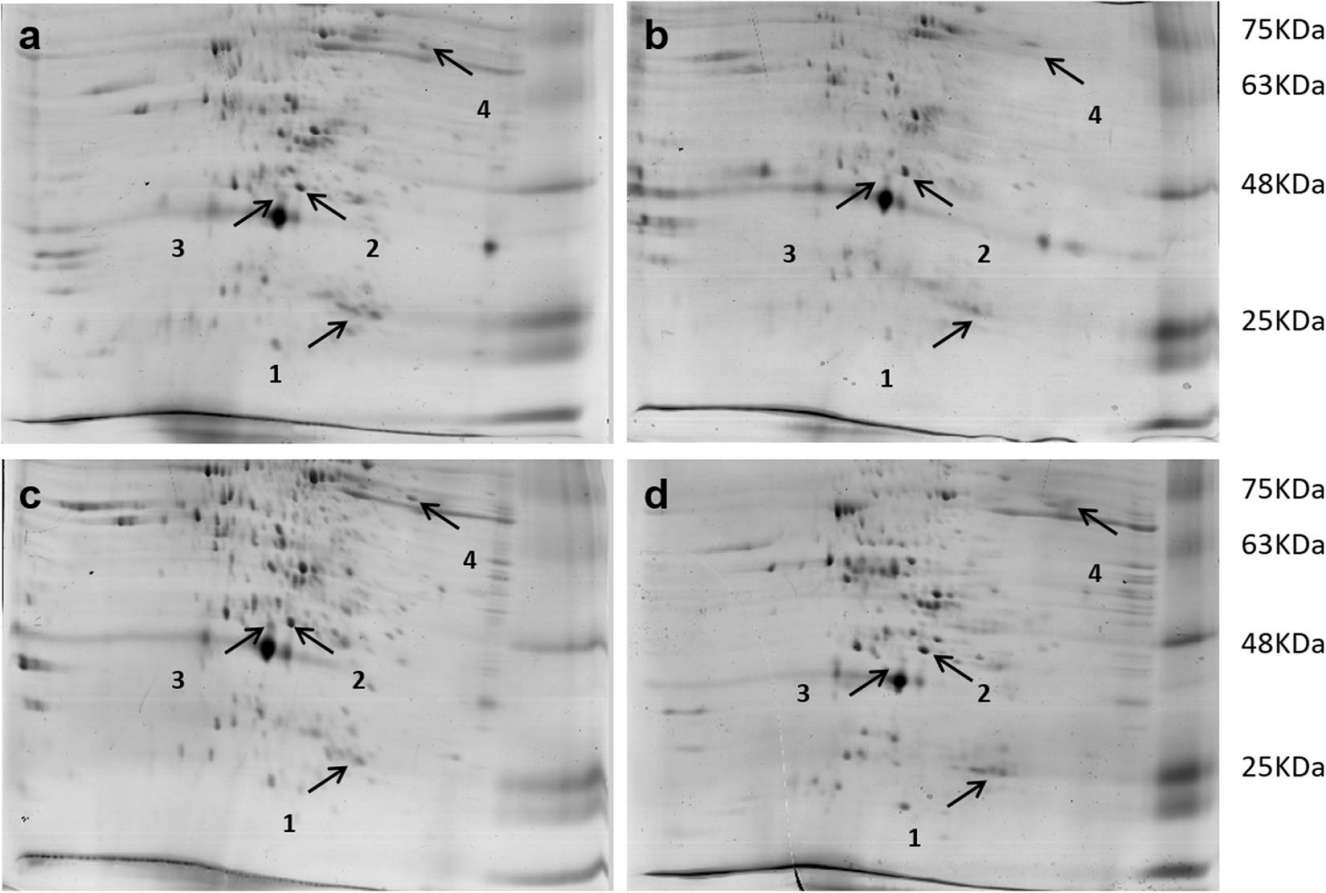
2-D gel electrophoresis profile of pyramided lines and parents.

**Figure 4:**
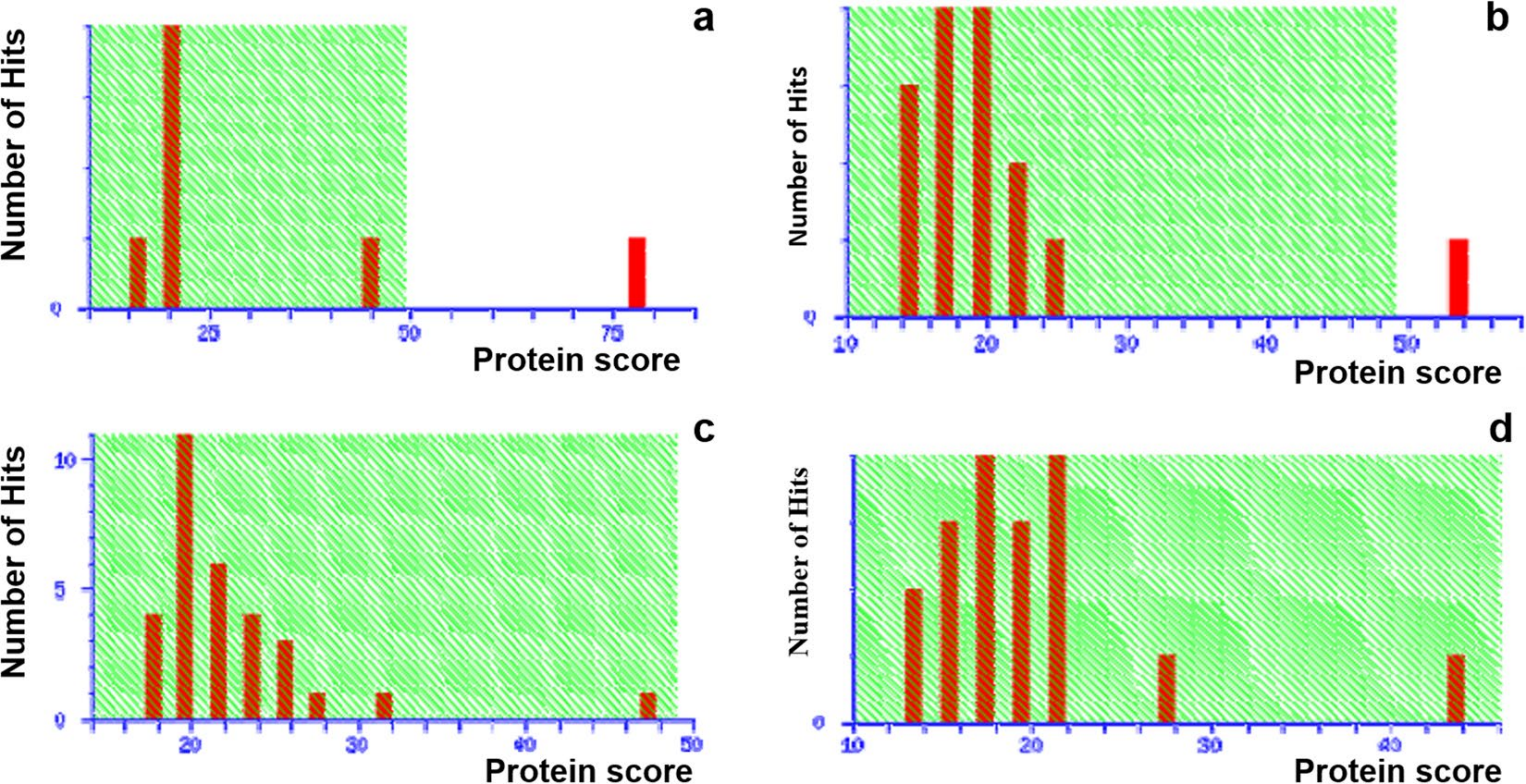
MALDI-TOF analysis of spots in recipient parent *Mushk Budji*/donor DHMAS 70Q 164-1b. (**a**) Alpha-amylase; (**b**) Triosephosphate isomerase; (**c**) 19 kDa globulin; d: S-(+)-linalool synthase.

Total of 171protein spots were detected by Image Master 2-D Platinum V.7.0 software (GE Healthcare, UK) among the two parents and lines SKUA-485-27-4-38-4, SKUA-485-27-4-40-6, of which 38 spots recorded a match count of three or four (Fig. 3). The match score for 21 protein spots (with match count of four) averaged at 0.38, 0.65, 0.67 and 0.72 for donor DHMAS 70Q 164-1b, SKUA-485-27-4-38-4, SKUA-485-27-4-40-6 and RP *Mushk Budji*. Based on the protein match score, the two PLs showed 90 and 93% similarity to RP *Mushk Budji* (Supplementary Fig. S7). Seventeen spots carried a valid match score for RP, SKUA-485-27-4-40-6 and SKUA-485-27-4-38-4, while these spots were absent in donor DHMAS 70Q 164-1b. Thirty-six protein spots were analyzed for peptide fingerprint using MALDI-TOF. Out of 36 spots we could find two peptides with significant match to SWISSPROT data base. These were identified as *Alpha-amylase* OS = *Oryza sativa* subsp Japonica GN = RASI PE = 1 SV = 2 and secondly, *Triosephosphate isomerase*, cytosolic OS = *Oryza sativa* subsp. The protein expression of *Alpha-amylase* was two-fold in *Mushk Budji* and backcross derived lines as compared to the donor DHMAS 70Q 164-1b (Fig. 4; Table 2; Supplementary Table S8). It had a score of 78 which is highly significant and also carried a protein sequence coverage of 22%. The theoretical pI and molecular mass was recorded at 8.66 and 21.689 kDa, respectively. *Triosephosphate isomerase*, recorded 16-fold change in SKUA-485-27-4-40-6 when compared to donor parent. The protein had a significant match score of 54 with pI and molecular weight of 5.38 and 27.274 kDa, respectively. The peptide showed coverage of 14% for 253 residue protein. Further, two more proteins scored near significance threshold and included, *19 kDa globulin* protein OS = *Oryza sativa* subsp. japonica GN = Os05g0499100PE = 1 SV = 2. It had a nominal mass of 21.497 kDa and a pI of 7.48. The sequence coverage of 21% was found for four matched peptides. The protein was up-regulated in *Mushk Budji* and derived backcross lines and recorded 3-8 fold change against DHMAS 70Q 164-1b. Protein namely, S-(+)-linalool synthase, chloroplastic OS = *Oryza sativa* subsp. Japonica GN = LIS PE = 2 SV = 1 was found with a score of 44. This featured with a nominal mass of 67.85 kDa and pI of 5.69. It had 10% coverage in 595 amino acid long protein and three matched peptides. The protein was upregulated in RP and derived lines (Table 2; Fig. 4; Supplementary Fig. S9).

**Table 2:** MALDI-TOF analysis of proteins showing differential expression between three-gene pyramids and donor lines.

**Spot ID**	**Protein name**	**Sequence coverage**	**Number of matched peptides**	**Mw (kDa) /pI**	Significance score*
33	Alpha-amylase	22%	3	21.689/8.66	78
47	Triosephosphate isomerase, cytosolic	14%	3	27.274/5.38	54
109	19 kDa globulin, mitochondrial	21%	4	21.497/7.48	49
133	S-(+)-linalool synthase, chloroplastic	10%	3	67.850/5.69	44

*Significance level is based on threshold score of 48 as per SWISSPROT database.

### Agronomic performance of the blast R-gene pyramided lines

**Figure 5:**
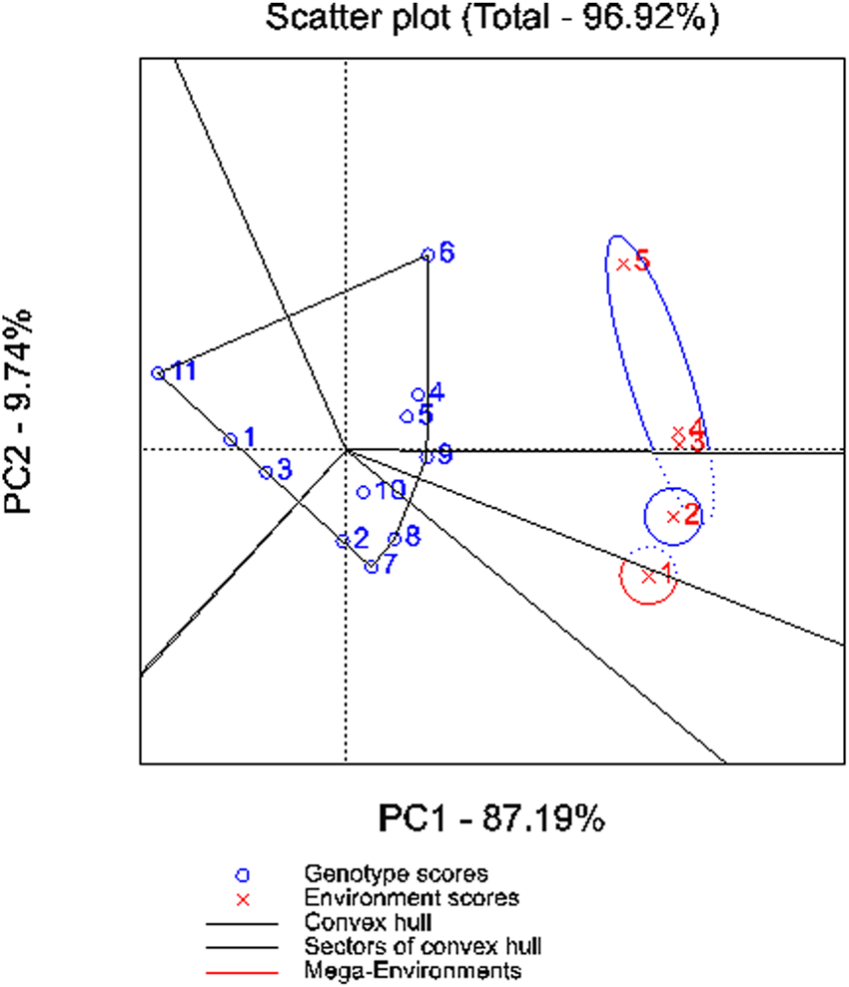
Biplot analysis of grain yield across locations. Blue marked: 1: SKUA-485-27-50-5-5; 2: SKUA-485-27-20-6-4, 3: SKUA-485-27-13-1-3;4: SKUA-485-27-86-10-4; 5:SKUA-485-27-77-6-2; 6:SKUA-485-27-47-4-1;7: SKUA-485-27-3-7-5;8: SKUA-485-27-4-38-4; 9:SKUA-485-27-4-40-6; 10: Mushk Budji; 11:DHMAS 70Q 164-1b; Red marked: 1: Sagam; 2: Pombay; 3: Khudwani; 4: Shalimar; 5: Budgam.

The biplot analysis helped us to partition total rice growing areas (represented here by E1, E2, E3, E4 and E5) of Kashmir into three mega-environments which favour ideal performance of *Mushk Budji* and the derived lines (refer section on methods). The environments E1 and E2 represented mega-environments-I and II, respectively, whereas E3, E4 and E5 constituted mega-environment-III. The ‘which-won-where’ biplot analysis revealed that genotype SKUA-485-27-47-4-1 performed better in non-traditional areas (E5). The BC_2_F_2:4_ PLs SKUA-485-27-20-6-4, SKUA-485-27-3-7-5 and SKUA-485-27-4-38-4 performed well in niche areas of *Mushk Budji* which constituted mega-environment E1. The genotype SKUA-485-27-4-40-6 was positioned on equality line between sub-environment (E3 and E4) and mega-environment-II. The donor genotype and SKUA-485-27-50-5-5 did not perform well in any of the locations. The Average Environment Coordination (AEC) view of the GGE biplot, which explains genotype comparisons on the basis of mean performance and stability across environments, helped to rank the genotypes on the AEC abscissa. The main effect of lines followed the sequence SKUA-485-27-86-10-4 > SKUA-485-27-4-38-4 > SKUA-485-27-4-40-6 > SKUA-485-27-77-6-2 = SKUA-485-27-3-7-5 > SKUA-485-27-20-6-4 > *Mushk Budji* > SKUA-485-27-13-1-3 > SKUA-485-27-50-5-5 > DHMAS 70Q 164-1b in order of high to low yield across environments. The genotypes SKUA-485-27-77-6-2 and SKUA-485-27-4-38-4like RP were found to be most stable genotypes across locations (Supplementary Tables S10, S11 and 12; Fig. 5).

### Grain and cooking quality of the blast R-gene pyramided lines

The donor and RP genotypes showed a difference of at least 1.3 mm with respect to milled rice length (KLBC) and 2.8 mm with respect to cooked kernel length (KLAC). The stringent phenotypic selection for these traits was employed along with background selection for grain type, KLAC, KLBC, KER and aroma followed by foreground selection for target genes in BC_1_F_n_ and BC_2_F_n_. A three-gene BC_1_F_1_ plant, SKUA-485-27 recorded KLBC and KLAC of 5.1 and 6.1 mm, respectively. It had poor KER as compared to RP *Mushk Budji*. The considerable improvement in mean KER (1.4) was realized in fifteen plants in BC_2_F_1_ derived from SKUA-485-27 (BC_1_F_1_). KLBC in SKUA-485-27 derived BC_2_F_1_s ranged from 5.0 to 5.6 mm and was similar to RP (5.1 mm) compared to donor (6.5 mm) parent. The PLs showed soft gel consistency in the range of 92 mm and 98 mm, similar to RP (115 mm) against donor parent which recorded hard GC (55 mm). The Alkali spreading value for three-gene PLs had a score of 4-5 same as *Mushk Budji* against a score of 6 for donor parent. Comparably low ASV in lines corresponds to higher gelatinization temperature than the RP parent. Amylose content for these lines ranged from 15.21 to 19.35% against 14.14% for RP *Mushk Budji* and 21.52% for DHMAS 170Q 164-1b. The PLs SKUA-485-27-4-38-4, SKUA-485-27-4-40-6 SKUA-485-27-3-7-5, SKUA-485-27-47-4-1 recorded KLBC, LBR, KLAC similar to RP *Mushk Budji*. KER for SKUA-485-27-4-38-4, SKUA-485-27-4-40-6, SKUA-485-27-3-7-5 was recorded at 1.47, 1.50 and 1.63, respectively. The lines showed aroma score of 2, similar to *Mushk Budji* (Supplementary Table S12; Supplementary Fig. S10).

### Disease reaction of the blast resistance gene pyramided lines

**Figure 6:**
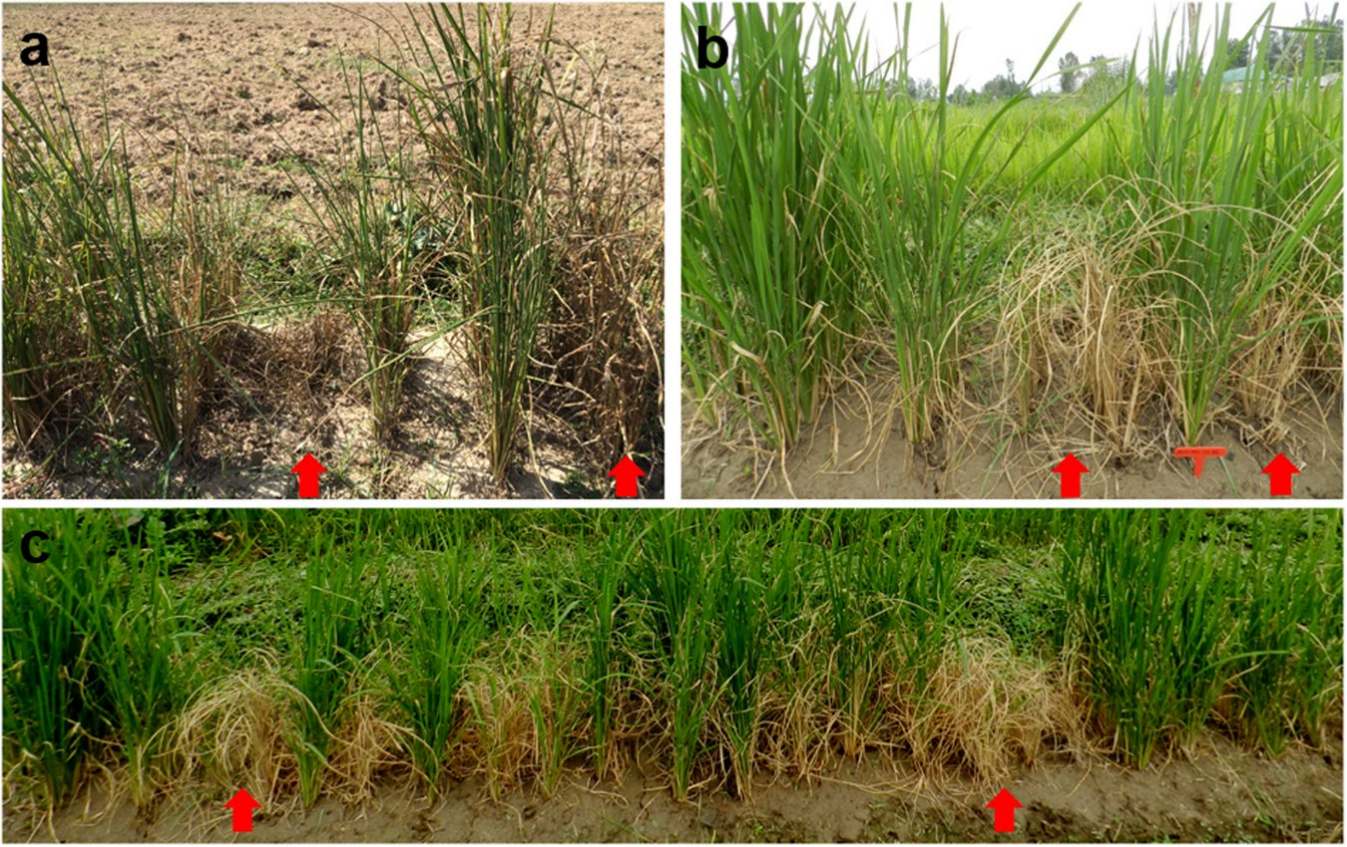
Disease reaction of three and two-gene pyramids under Uniform Blast Nursery. (**a**) Disease reaction recorded at location Sagam; (**b**) Magnified view of boxed portion in c; (**c**) Disease reaction recorded at location Khudwani; L1: SKUA-485-27-4-38-4; L2: SKUA-485-27-4-40-6; L3:SKUA-485-27-86-10-4; L4: SKUA-485-27-13-1-3; L5: SKUA-485-27-50-5-5; L6: SKUA-485-27-78-3-4; L7: SKUA-485-27-20-6-4; L8: SKUA-485-27-47-4-1; L9: SKUA-485-27-77-6-2; L10: SKUA-485-27-3-7-5; L11: SKUA-485-27-20-8-10; L12: SKUA-485-27-47-5-3; L13: SKUA-485-27-86-6-1; L14: SKUA-485-27-61-2-5; L15: SKUA-485-27-64-3-8; MB: Mushk Budji (Recurrent parent); K: Kohsar (Susceptible check).

The nine PLs were evaluated for blast disease reaction using four diagnostic isolates of *M. oryzae* under controlled conditions in presence of donor and RP checks. The isolate Mo-ei-MBI-2, characterized for its specificity to genes *Pi54* and *Pita*, whereas isolate Mo-nwi-kash-32, avirulent to genes *Pi54* and *Pi1* could not overcome the BC_2_F_2:4_ PLs carrying *Pi54*+*Pi1*+*Pita*. Most of the BC_1_F_3:5_ lines expressed hyper-sensitive response against isolates SKUA-Mo-3 and SKUA-Mo-9, while the RP *Mushk Budji* was highly susceptible (Table 3; Supplementary Figs S15–18). A same set of lines was tested in Uniform Blast Nursery at hot spot locations of *Sagam, Khudwani, Pombay* and *Shalimar* in Kashmir valley for disease reaction under multiple isolate conditions. All the lines expressed resistance response to prevalent isolates while, the RP *Mushk Budji* planted as check succumbed to the disease at all the locations. SKUA-485-27-50-5-5 showed moderate susceptibility at *Sagam* and *Pombay*. The high level of disease susceptibility was noted for *Mushk Budji* in its niche area of cultivation i.e. *Sagam* where all the lines barring one showed hypersensitive reaction response (Supplementary Table S13; Fig. 6).

**Table 3:** Disease reaction against specific *Magnaporthe oryzae* isolates under controlled conditions.

**S. No**.	**Plant ID**	**Gene combination**	**Isolates**			
			**Mo-ei-MBI-2**	**Mo-nwi-kash-32**	**SKUA-Mo-3**	**SKUA-Mo-9**
1	SKUA-485-27-50-5-5	*Pi54*+*Pi1*+*Pita*	1	1	2	2
2	SKUA-485-27-20-6-4	*Pi54*+*Pi1*+*Pita*	0	0	1	1
3	SKUA-485-27-13-1-3	*Pi54*+*Pi1*	0	0	2	2
4	SKUA-485-27-86-10-4	*Pi1*+*Pita*	0	0	0	1
5	SKUA-485-27-77-6-2	*Pi54*+*Pi1*+*Pita*	1	0	2	2
6	SKUA-485-27-47-4-1	*Pi54*+*Pi1*	1	0	1	1
7	SKUA-485-27-3-7-5	*Pi54*+*Pi1*+*Pita*	0	0	0	1
8	SKUA-485-27-4-38-4	*Pi54*+*Pi1*+*Pita*	0	0	1	0
9	SKUA-485-27-4-40-6	*Pi54*+*Pi1*+*Pita*	1	1	0	1
10	*Mushk Budji*	—	5	5	5	5
11	DHMAS 70Q 164-1b	*Pi54*+*Pi1*+*Pita*	0	0	0	0

Leaf blast scoring was performed as per 0-5 scale^21^; Score 0-2: R; 3-5: S.

**Table 4:** SNP loci known for their relation to important agronomic traits and their recovery in backcross derived lines.

**Affy SNP ID**	**Locus ID**	**Chr**	**Gene**	**R**	**D**	**1**	**2**	**3**	**4**	**5**	**6**	**7**	**8**	**Trait**	**Function**
Affx-93217388	LOC_Os01g62410	1	*MYB3R2*	A	B	A	A	B	A	A	A	A	B	Chilling Stress	R1R2R3 MYB transcription factor^22^
Affx-93211543	LOC_Os03g52460	3	*AGPL1*	A	B	A	A	A	A	A	A	A	A	Grain quality, amylose content and viscosity (starch biosynthesis)	Glucose-1-phosphate adenylyl transferase^23^
Affx-93254079	LOC_Os06g06560	6	*SSS1*	A	B	A	A	A	A	A	A	A	B	Grain yield and grain quality	Soluble starch tarch synthase 1^24^
Affx-93222660	LOC_Os06g51084	6	*SBE1*	A	B	H	H	A	H	H	H	H	A	Cooking quality	1,4-alpha-glucan branching enzyme^25^
Affx-93221425	LOC_Os11g11580	11	*NAC5*	A	B	A	A	H	H	B	A	H	A	Cold and drought tolerance	NB-ARC domain containing protein^26^

R: Mushk Budji; D: DHMAS 70Q 164-1b; 1-8: SKUA-485-27-4-38-4; SKUA-485-27-4-40-6; SKUA-485-27-3-7-5;SKUA-485-27-13-1-3; SKUA-485-27-20-6-4; SKUA-485-27-47-4-1; SKUA-485-27-77-6-2; SKUA-485-27-86-10-4; A: RP allele; B: Donor allele; H: Heterozygous.

## Discussion

Although conventional breeding assumes RPG recovery at the rate of 1 − (1/2)^n+1^ for every ‘n’ generations of backcrossing^27^, Marker-assisted backcross breeding^28^ (MAB) approach helped us to pyramid three dominant blast resistance genes (*Pi54, Pi1* and *Pita*) along with rapid RPG recovery as early as in BC_2_F_3_ generation. Pyramiding multiple genes in a single variety based on phenotyping alone would be near impossible due to difficulty in estimating resistance response of component genes individually^29^. Further, the conduct of detailed pathotyping assay at various backcross generations can be avoided with the use of molecular markers^30^. Marker-assisted foreground selection was carried out using the markers Pi54 MAS, RM224 and a marker pair YL 155/87 and YL 155/83, to select for the genes *Pi54*, *Pi1* and *Pita*, respectively. Of these, Pi54 MAS, a gene based InDel marker amplifies 216 bp fragment specific to *Pi54* resistance and 359 bp allele for susceptible plants^31^. RM224 is a linked SSR marker that is located at 0 cM from gene *Pi1*^32^. The gene *Pita* is located at centromeric region of chromosome 12 and was selected using gene based coupling-repulsion marker pair YL 155/87 and YL 155/83 which target transcription start site of the gene^33^. The use of gene based markers allows transfer of gene of interest with high precision and accuracy. Theoretical expectations in using linked marker for foreground section are such that for 10 cM distance between flanking markers and gene of interest, there lies a 0.024 probability of losing the gene after single generation which goes up to 0.1182 after five generations. That means maintenance of lines using same marker at a considerable distance from gene would rather not be practicable in long term after release of the product.

A single three-gene (*Pi54*+*Pi1*+*Pita*) donor, DHMAS 70Q 164-1b originating from a Vietnamese *indica* rice cultivar Tetep^34^, was used as male parent in a cross with *Mushk Budji*. The genes selected here have been known for effectiveness across various locations inside the target region^6,13^. The plants in BC_1_F_1_ showed wide range in spikelet sterility (3.8 to 92.0%) that may be attributed to genetic divergence between donor and recurrent parents^35,36^. The selection for recombinants having better spikelet fertility (SF) was achieved directly through selection for fertile panicles and indirectly by means of selection against heading-date as has been supported elsewhere^37^. The mean grain yield per plant in SKUA-485-27 showed increase from 20.5 g in BC_1_F_1_ to 22.0 g in BC_2_F_1_. The marker-assisted background selection, as a method to accelerate the RPG recovery through selection of RP alleles at large number of loci, as per Frisch *et al*.^38^, leads to selection response ‘R’ which is decided by the multiplicative action of selection intensity (i), standard deviation of RPG (σ) and correlation between the proportion of RP alleles at marker loci and the proportion of RP alleles across the whole genome (r). Therefore, one way to account for the proportion of genome besides those of marker loci, would be to carry selection for the easily observable (phenotypic) traits that would enhance ‘r’ in above equation. Phenotypic selection was also performed in segregating backcrosses to recover lines with grain and kernel traits with high similarity to *Mushk Budji*.

The PLs in BC_1_F_3:4_ (SKUA-485-27-47-4-1 and SKUA-485-27-77-6-2) and BC_2_F_2:3_ (SKUA-485-27-4-38-4, SKUA-485-27-4-40-6, SKUA-485-27-3-7-5), confirmed for homozygosity at target genes, were analyzed for background genome recovery that ranged from 82.69 to 91.03%. The preferential selection of individuals based on RPG recovery on carrier chromosomes was avoided as supposedly it would have resulted in lower overall RPG content^39^. Here, the stringent selection on carrier chromosomes if performed, might have resulted in reduced selection pressure on non-carrier chromosomes which form the major part of the genome. Also, the two-stage selection process is said to be superior to three-and four-stage selection process in breeding programs aimed up to BC_1_ and BC_2_^40^.

The final set of PLs showed RP allele at BADH2 locus at chromosome 8 that correlated well with the phenotype. These lines also carried RP allele at Wx locus on chromosome 6 and 8 and were phenotypically similar to RP *Mushk Budji*. The lines SKUA-485-27-86, SKUA-485-27-70 and SKUA-485-27-47 showed *Mushk Budji* allele at markers PNK7 and PNK10, which are linked to BADH1 locus for aroma on chromosome 4. The lines with maximum recovery and better plant and grain type were confirmed to have recovered at SSR loci RM6666 on chromosome 1, which is linked to the QTL for cooking quality^41^. The chromosomes 1, 3 and 4 recorded better recovery for recipient parent genome. The chromosome 1 carries gene for plant height and the traits related to grain dimensions. The genes for KER and LBR are located on chromosomes 3 and 4. The quick recovery in BC_1_F_2_ and subsequent selfing generations towards shorter grain length and KLBC similar to *Mushk Budji*, shall be explained by dominant nature of loss of function QTLs responsible for shorter grain length such as GS-3, qGL-3, etc., over those responsible for fine and long grained phenotype caused by recessive alleles at these loci^42^. Marker-assisted background selection with the help of 15 SSR markers on chromosome 11 helped to minimize the linkage drag around gene *Pi54* within 2.7 Mb between markers RM3917 and RM26963. On the same chromosome the gene *Pi1* was delimited to 1.2 Mb region. Similarly, the segment carrying *Pita* was narrowed down to 4.1 Mb. In conventional breeding programs expected drag around target locus is 79 and 63 cM in BC_1_F_1_ and BC_2_F_1_, respectively^43^. So to our advantage, the background markers helped to reduce the drag down to 4 to 16 cM in BC_2_F_1_ PLs. The results are in line with expectations as per simulated experiments carried out by Frisch *et al*.^44^, where it was concluded that 71 and 86% of genome would be recovered in BC_1_F_1_ and BC_2_F_1_ for a population having 20 individuals. Singh *et al*.^20^ incorporated *Pi54* and *Piz5* in PRR78 background and achieved RPG of 89.17 and 87.88%, respectively. Gopalakrishnan *et al*.^45^ used foreground and background selection to develop improved PB 1 with *xa13*and *Xa21* with minimum linkage drag above 1.3 cM.

Specifically, few SNP loci that have been reported to be linked to the traits of agronomic importance, rice quality and tolerance to cold stress, were scored for their recovery in BC_2_F_4_ lines and carried RP alleles (Table 4). Overall, the background analysis of PLs using 50 K SNP array revealed low RPG recovery compared to the estimate based on SSR markers. Clearly, the recombination events were resolved efficiently through high density SNPs. Overestimation of background genome recovery using SSR markers has been a feature reported previously^9^. Further, the estimate of RPG similarity (%) rather than RPG recovery (%) may be suggested to be the better option for evaluation of background genome content in backcross derived lines. The genomic proximity between donor and RP based on RPG similarity (%) may help to decide on the necessity of further advancement of backcross generations in a more realistic manner. As in our case, RP and donor share 56.2% genome similarity, so that two backcrosses were sufficient to yield more than 90% similarity among lines and RP.

Besides marker based estimation of RPG recovery, this study reports the evaluation of PLs for recovery on the basis of protein profile. 2-D gel electrophoresis detected 171 protein spots which showed differential expression pattern among selected backcross derived lines and parents. The selected 38 clearly differentiated spots showing match score of 4 were analyzed and revealed an average similarity of 97, 79 and 68% for SKUA-485-27-4-38-4, SKUA-485-27-4-40-6 and donor DHMAS 70Q 164-1b, respectively, against RP *Mushk Budji*. Therefore, in general, the PLs, not only had high genome recovery based on SNP and SSR markers, but also for matrix of proteins in 2-D profile. Also, 16 spots present in RP and lines were altogether absent in donor DHMAS 70Q 164-1b. MALDI-TOF analysis for peptide fingerprint was performed with 36 protein spots of which, two peptides had significant match to SWISSPROT data base. These included *Alpha-amylase* OS = *Oryza sativa* subsp Japonica GN = RASI PE = 1 SV = 2 that had a significant score of 78 with a protein sequence coverage of 22%. The protein expression was almost two-fold in *Mushk Budji* and backcross derived lines as compared to the donor DHMAS 70Q 164-1b. The theoretical pI and molecular mass was recorded at 8.66 and 21.689 kDa, respectively. *Alpha-amylase* is responsible for breakdown of starch in rice. The expression of Alpha amylase have shown differential expression across varieties^46^. The 19 kDa *globulin* protein OS = *Oryza sativa* subsp. japonica GN = Os05g0499100PE = 1 SV = 2 had a nominal mass of 21.497 kDa and a pI of 7.48. The sequence coverage of 21% was found for 186 amino acid residue protein. Globulin is an important storage protein concentrated to bran layer of brown rice compared to glutelin that is mainly confined to endosperm. The protein was upregulated in *Mushk Budji* and derived backcross lines as compared to DHMAS 70Q 164-1b. A 67.85 kDa protein, S-(+)-linalool synthase, chloroplastic OS = *Oryza sativa* subsp. Japonica GN = LIS PE = 2 SV = 1, at pI of 5.69 was upregulated in RP and derived lines and downregulated in donor with 10% coverage in 595 amino acid chain. The protein is reported to be involved in monoterpene biosynthesis. The major product is S-(+)-linalool that is induced by jasmonate in response to *Xanthomonas oryzae*^47^. However, role in rice blast resistance has not been elucidated.

GGE biplot analysis^48^ combines genotype (G) main effects and genotype x environment (GE) component to work out the genotype performance and the helps to designate the mega-environments supporting such genotypes. This is a robust technique to mark genotypes in a which-won-where fashion and helped us to understand about performance of derived lines within and outside the target regions.

The final set of selected PLs were screened under artificial conditions and showed resistance against the four *M. oryzae* isolates in presence of RP check that succumbed to the disease. The virulence analysis carried out initially using LTH background differential set for isolates SKUA-Mo-3 and SKUA-Mo-9 confirmed them to possess *Avr-Pita*. Mo-ei-MBI-2 and Mo-nwi-kash-32 were procured from Dr. U. D. Singh, Indian Agricultural Research Institute, New Delhi, India and could detect the genes *Pi54, Pita* and *Pi54, Pi1*, respectively. The isolate Mo-nwi-kash-32 was collected from RP *Mushk Budji* and could accurately confirm the resistance carried by lines. Further, these lines were tested at five blast hot spot locations within *Kashmir* valley. All the lines expressed resistance response to prevalent isolates while, the RP *Mushk Budji* planted as check succumbed to the disease at each of these locations. This substantiates our choice of participating genes in constituting PLs and their suitability to be released as cultivars in the traditional *Mushk Budji* growing areas.

Marker-assisted selection and high throughput validation of RPG recovery lead to the development of PLs of *Mushk Budji* carrying genes for blast resistance. The lines developed here are set for their release as improved versions of *Mushk Budji* for commercial cultivation in farmers’ fields. This is a rare report on improvement of an aromatic rice landrace for resistance to disease like blast. Though short and medium grained traditional rice varieties comprise 4.4% of the total rice cultivars grown by farmers across the globe, and global scented rice market is growing at 12% per annum, a holistic approach needs to be adopted for conservation, promotion and genetic enhancement of such valuable rice cultivars.

## Materials and Methods

### Plant materials

*Mushk Budji*, a popular short-grained aromatic rice landrace of Jammu and Kashmir, India, which is highly susceptible to blast disease (Supplementary Fig. S11), was used as RP and crossed as a female to a blast resistance donor parent (DP) *DHMAS 70Q 164-1b*. The donor parent is a doubled haploid line obtained from the cross *HPU741*/*Tetep* and harbors three blast resistance genes, *Pi54, Pi1*and *Pita*.

### Marker-assisted backcross breeding

From the *Mushk Budji*/ DHMAS 70Q 164-1b cross, a single F_1_ plant with confirmed hybridity was backcrossed with *Mushk Budji* to generate BC_1_F_1_ plants. Subsequently, the selected BC_1_F_1_ plant was crossed to the RP to generate BC_2_F_1_ and advanced further through selfing by following a marker-assisted backcross breeding (MABB) scheme (Supplementary Fig. S1). The scheme comprised of a four-step selection strategy in each backcross generation: (1) foreground selection for the target genes using gene-based/linked DNA markers; (2) recombinant selection using DNA markers flanking the respective target genes; (3) background selection using polymorphic DNA markers, (4) stringent phenotypic selection for agro-morphological traits, grain dimension, cooking quality and aroma to accelerate the recurrent parent phenome (RPP) recovery. The marker-assisted foreground selection for genes *Pita* and *Pi54* was carried out using coupling-repulsion pair of gene-based markers YL155/YL87//YL155/87^33^ and Pi54MAS^31^, respectively. The selection for the gene *Pi1*, was carried out using gene-linked marker RM224^32^ (Supplementary Table S14). The marker-assisted background selection was conducted using genome wide SSR markers. The RP *Mushk Budji* and DP *DHMAS 70Q 164-1b* were surveyed for polymorphism with 278 genome wide SSR/genic markers. The marker information was retrieved from http://www.gramene.org and published literature^49^. The extent of RPG recovery was calculated as per *Khanna et al*.^9^. The primers were custom synthesized by Sigma Technologies Inc., USA. The RPG recovery was graphically represented using Graphical Geno Typing (GGT 2.0) software^50^.

### DNA extraction and PCR amplification of SSR and STS markers

DNA was extracted from leaves using CTAB (Cetyl-Tri Methyl Ammonium Bromide) procedure described by Murray and Thompson^51^. Polymerase chain reaction (PCR) was performed in a thermal cycler (TaKaRa, Shiga, Japan) in PCR reaction mix containing 25 ng of genomic DNA, 1 ul of 10× PCR buffer (10 mM Tris, pH 8.4, 50 mM KCl, 1.8 mM MgCl_2_), 2 mM dNTPs (MBI, Fermentas, Lithuania, USA), 5 pmol each of the forward and reverse primers and 3 U of Taq DNA polymerase (MBI, Fermentas, Lithuania, USA) in a reaction volume of 10 μl. The PCR program for markers Pi54MAS and RM224 was: initial denaturation at 94 °C for 5 min; followed by 35 cycles of denaturation at 94 °C for 30 s, annealing at 55 °C for 30 s, and extension at 72 °C for 1 min; and a final extension at 72 °C for 7 min. Similar program was used for the marker YL155/87 but with doubling the times for denaturation, annealing and extension steps. The PCR amplicons were resolved by electrophoresis in 1.5% agarose for Pi54MAS and YL155/87 and 3.5% agarose gel for RM224) and visualized and documented using gel documentation system (Bio-Rad Laboratories Inc., USA).

### Analysis of background recovery using 50 K SNP genotyping array

A high resolution analysis of background recovery was done on a set of eight selected BC_1_F_3:4_ and BC_2_F_2:3_ lines in comparison with the RP *Mushk Budji* and the DP DHMAS 70 Q 164-1b using ‘OsSNPnks’ 50 K Axiom® 2.0 SNP array^52^. High quality genomic DNA for the assay was quantified using Nanodrop spectrophotometer and concentration was adjusted to 20 ng/µl with OD_260/280_ values in the range of 1.8–2.0.

For target probe preparation, 20 μl of gDNA was used for each DNA sample at a concentration of 10 ng/μL (for a total 200 ng DNA in 20 μl) based on Affymetrix Axiom® 2.0 Assay Manual. DNA amplification, fragmentation, chip hybridization, DNA ligation and signal amplification were performed using the Affymetrix Axiom® 2.0 Assay Manual Target Prep Protocol QRC (P/N 702990). Staining and scanning were performed on the GeneTitan® Multi-Channel Instrument according to the manufacturer’s procedure (http://media.affymetrix.com). The assay included 50,051 high quality non-redundant SNPs mostly representing single-copy (SC) genes from whole rice genome with an average interval of 7.45 Kb between SNPs.

### 2-D SDS-PAGE MALDI-TOF-TOF analysis of seed proteome

Two most promising MABB derived lines, SKUA-485-27-4-38-4 and SKUA-485-27-4-40-6, along with the RP and DP were analyzed for their seed protein composition using 2-D SDS-PAGE MALDI-TOF-TOF. SYSTEM. The 2 g of dehulled grains of samples were finely grounded using liquid nitrogen with the help of pre-chilled mortar and pestle. The powdered sample was taken in a 1.5 ml centrifuge tube and 500 µl Phosphate Buffer (0.1 M, pH7.5) was added. The samples were vortexed and centrifuged at 14000 rpm 15 min. The supernatant was transferred to a fresh tube containing 10% (w/v) TCA in acetone with 0.07% (v/v) 2-ME for protein precipitation. The protein pellet obtained after centrifugation at 14000 rpm at 4 °C for 15 min was washed thrice with chilled acetone supplemented with 2-ME (0.07%), EDTA (2 mM) and 1 tablet of complete EDTA free protease inhibitor (carrying Pancreas extract, Thermolysin, Chymotrypsin, Trypsin, Papain with 0.02, 0.0005, 0.002, 0.02, 0.33 mg/ml, respectively). Final washing was done with pure acetone. Pellet was kept at −80 °C for overnight. Acetone-free pellet was dissolved in rehydration buffer (8 M Urea, 20 mM DTT, 2% w/v CHAPS). Protein quantification was done according to Bradford^53^ and 250 µg of protein containing IPG buffer and DTT was loaded in rehydration tray. Immobilized protein gradient (IPG) strips (pH3–10, 13 cm, GE Healthcare, UK) were rehydrated overnight. IEF was carried out using Ettan IPGPhor3 (GE Healthcare, UK) with standardized programme. Second dimension electrophoresis was carried out using Hoefer SE600 Ruby electrophoresis unit (GE Healthcare, UK) at 40 mA/gel for two and a half hours at 25 °C. The gels were stained overnight with CBB solution as described^39^ and then destained with 0.5 M NaCl solution^54^. All 2-D CBB stained gels were scanned with GE Image Scanner III at 300dpi and analyzed using Image Master 2-D Platinum V.7.0 software (GE Healthcare, UK).

Protein spots were excised from preparative polyacrylamide gels that had been stained with Comassie Brilliant Blue G-250and each gel fragment was immersed in purified water and sonicated twice for 10 min each time at 50 W and 20 kHz. Subsequently, the gel pieces were destained with 50 mM ammonium bicarbonate and an equivalent volume of 50% acetonitrile, followed by sequential washing with 25 mM ammonium bicarbonate, 50% acetonitrile and 100% acetonitrile, respectively. After lyophilization, the gel fragments were rehydrated in digestion buffer containing 25 mM NH_4_HCO_3_ and 10 ng of trypsin/L (Promega, Madison, WI, USA) at 4 °C. Proteins were digested with trypsin and MS analysis was conducted with a Matrix-assisted laser desorption and ionization-time of flight (MALDI-TOF) mass spectrometer 4700 Proteomic Analyzer (Bruker, Germany).

### Multi-location testing of pyramided lines for grain yield performance

The PLs in BC_1_F_3:5_ (SKUA-485-27-50-5-5, SKUA-485-27-20-6-4, SKUA-485-27-13-1-3, SKUA-485-27-86-10-4, SKUA-485-27-77-6-2, SKUA-485-27-47-4-1) and BC_2_F_2:4_ (SKUA-485-27-3-7-5, SKUA-485-27-4-38-4, SKUA-485-27-4-40-6) were sown across five different locations namely, Sagam (E1, 1900 m), Pombay (E2, 1900 m), Khudwani (E3, 1560 m), Shalimar (E4, 1520 m), Budgam (E5, 1480 m). The lines along with two parents, DHMAS 70Q 164-1b and *Mushk Budji*, were grown in Randomized Block Design with three replications under irrigated ecology. Similar management was followed at all the locations and observations were recorded on traits PH, NT, GP, SF, SW, DM and GY. GGE Biplot analysis was carried out to workout genotype relation across different environments with respect to grain yield performance. A “which-won-where” view of GGE biplot was used to characterize the genotypes for their agronomic performance and to demarcate distinct mega-environments which suit them. For our purpose, the biplot was explained using a polygon, spread across the coordinates, with perpendicular lines, called equality lines, drawn onto its sides. The lines thus create the sectors which hold the particular environments. Genotypes located on the vertices of the polygon were regarded as the best performers within the sector^55^. The AEC view of the GGE biplot, which explains genotype comparisons on the basis of mean performance and stability across environments, was drawn to rank the genotypes on the AEC abscissa. The GGE biplot analysis was conducted using the software Genstat v.12.

### Evaluation for blast disease resistance under controlled conditions

The isolates collected from *Mushk Budji* were used for inoculation for screening the gene PLs for resistance to rice blast. The genes *Pi54, Pi1 and Pita* were tested using isolates Mo-nwi-kash-32 and Mo-ei-MBI-2 kindly provided by Division of Plant Pathology, IARI New Delhi. The seedlings were inoculated at three-leaf stage by spraying 50 ml of spore suspension (~5 × 10^4^ conidia ml^−1^), and incubated in growth chambers for 24 h in dark at 26–27 °C. The seedlings were sprayed with water after every 6–7 h to maintain the humidity for 4–5 days to facilitate the penetration by the fungus and disease establishment. The disease was scored after 7 days of inoculation using the scale given by Mackill and Bonman^21^.

### Evaluation for blast disease resistance under field conditions

The pyramids were also screened in Uniform Blast Nursery at five hot spot locations in Jammu and Kashmir, viz, *Sagam, Pombay, Khudwani, Shalimar* and *Budgam*. A 50-cm row each of the gene PLs along with the RP and DP controls was planted in a raised bed nursery with a row to row spacing of 10 cm. To ensure uniform spread of disease, a row of susceptible check was planted after every five rows as well as on the borders. The disease evaluation was done on 0–9 Standard Evaluation Scale of IRRI^56^. The lines with 0–3 score were considered as resistant, those with score of 4–5 were regarded as moderately resistant, those having score of 6–7 were treated as moderately susceptible and those with score of 8–9 were considered to be susceptible.

## Electronic supplementary material

Supplementary Tables and Figures

Data set 1
