# Supplementary material for: Marker-assisted introgression of three dominant blast resistance genes into an aromatic rice cultivar Mushk Budji
Source: Sci Rep. 2018 Mar 6;8:4091. doi: 10.1038/s41598-018-22246-4 (PMC5840290; doi:10.1038/s41598-018-22246-4)
Supplement: Supplementary file 1 — Supplementary Tables and Figures [file 41598_2018_22246_MOESM1_ESM.pdf]

Marker-assisted introgression of three dominant blast resistance genes into an aromatic rice cultivar *Mushk Budji*

Gazala Hassan Khan[1], Asif Bashir Shikari[1*], Rakesh Vaishnavi[2], Sofi Najeeb[3], Bilal A. Padder[4], Zahoor A. Bhat[4], Ghulam A. Parray[3], Mohammad Ashraf Bhat[5], Ram Kumar[6] and Nagendra K. Singh[6]

[1]Division of Plant Biotechnology, Sher-e-Kashmir University of Agricultural Sciences and Technology of Kashmir, Shalimar Campus, Srinagar, J & K, India

[2] DARS, Old Airport Road, Sher-e-Kashmir University of Agricultural Sciences and Technology of Kashmir, Budgam, J & K, India

[3]Mountain Research Centre for Field Crops, Sher-e-Kashmir University of Agricultural Sciences and Technology of Kashmir, Khudwani, J & K, India

[4]Division of Plant Pathology, Sher-e-Kashmir University of Agricultural Sciences and Technology of Kashmir, Shalimar Campus, Srinagar, J & K, India

[5]Division of Genetics and Plant Breeding, Sher-e-Kashmir University of Agricultural Sciences and Technology of Kashmir, FOA, Wadura, Sopore, Baramullah, J&K, India

[6]ICAR- National Research Centre on Plant Biotechnology, Lal Bahadur Shastri Building, Pusa Campus, New Delhi, India

***Corresponding author E-mail: asifshikari@gmail.com**

Supplementary Table S1: Agronomic performance of three-gene BC_1F_1 derived BC_1F_2 plants of *Mushk Budji* / DHMAS 70Q 164-1b

Plant ID	*Pi54*	*Pi1*	*Pita*	PH	NT	PL	SP	SF	GY
SKUA-485-27-13	B	B	A	120	20	23	95	68.42	11.9
SKUA-485-27-20	B	B	B	104	12	21	115	72.2	8.6
SKUA-485-27-32	B	B	B	115	15	23	152	88.8	14.3
SKUA-485-27-43	B	A	B	133	21	24	175	69.1	23.0
SKUA-485-27-47	B	B	A	118	13	22	92	89.1	7.5
SKUA-485-27-50	B	B	B	123	12	19	93	97.8	7.0
SKUA-485-27-56	B	B	B	132	11	22	100	89.0	6.9
SKUA-485-27-61	B	B	B	140	15	21	85	82.9	8.0
SKUA-485-27-64	B	B	A	135	12	23	174	64.9	13.1
SKUA-485-27-70	B	B	B	117	21	21	197	84.3	25.9
SKUA-485-27-77	B	B	B	127	20	21	122	89.2	15.3
SKUA-485-27-78	B	B	B	103	16	21	112	85.2	11.2
SKUA-485-27-81	B	B	B	120	17	21	111	81.4	11.8
SKUA-485-27-86	A	B	B	113	20	23	146	80.1	18.3
**Mean**				**121.4 ±3.0**	**16.1 ±1.0**	**21.8 ±0.4**	**126.4 ±9.6**	**81.6 ±2.6**	**13.0 ±1.6**
**MAX**				**140.0**	**21.0**	**24.0**	**197.0**	**97.8**	**25.9**
**MIN**				**103.0**	**11.0**	**19.0**	**85.0**	**64.9**	**6.9**
**DHMAS 70Q 164-1b**	**B**	**B**	**B**	**107**	**15.6**	**20.5**	**124.7**	**65.2**	**12.2**
Mushk Budji	**A**	**A**	**A**	**127**	**15.4**	**19.6**	**114**	**86.1**	**11.0**

PH: Plant Height (cm), NT: Number of effective Tillers/ plant, PL: Panicle Length (cm), SP: Spikelets/ Plant, SF: Spikelet Fertility (%), GY: Grain Yield/ plant (g), A: Recipient (susceptible) parent allele, B: Donor (resistant) Parent allele

Supplementary Table S2: Kernel traits and cooking quality of three-gene BC_1F_1 derived BC_1F_2 plants of *Mushk Budji*/ DHMAS 70Q 164-1b

**Plant ID**	***Pi54***	***Pi1***	***Pita***	**KLBC**	**LBR**	**KLAC**	**KER**	**Aroma**
SKUA-485-27-13	B	B	A	4.50	1.8	7.00	1.56	1
SKUA-485-27-20	B	B	B	5.0	2.5	8.2	1.64	1
SKUA-485-27-32	B	B	B	4.5	1.8	7.0	1.56	2
SKUA-485-27-43	B	A	B	5.0	2.0	7.6	1.52	0
SKUA-485-27-47	B	B	A	4.5	1.8	7.0	1.56	2
SKUA-485-27-50	B	B	B	4.5	1.8	7.0	1.56	1
SKUA-485-27-56	B	B	B	5.0	2.5	7.8	1.56	2
SKUA-485-27-61	B	B	B	5.0	2.0	7.5	1.50	1
SKUA-485-27-64	B	B	A	4.4	1.8	7.0	1.59	1
SKUA-485-27-70	B	B	B	5.0	2.1	7.0	1.40	1
SKUA-485-27-77	B	B	B	4.5	2.3	8.4	1.87	1
SKUA-485-27-78	B	B	B	4.5	1.8	7.0	1.56	1
SKUA-485-27-81	B	B	B	4.5	1.9	7.0	1.56	1
SKUA-485-27-86	A	B	B	5.0	2.0	8.6	1.72	1
**Mean**				**4.7±0.1**	**2.0±0.1**	**7.4±0.2**	**1.58±0.1**	
**MAX**				**5.0**	**2.0**	**8.6**	**1.72**	
**MIN**				**4.4**	**2.2**	**7.0**	**1.59**	
**DHMAS 70Q 164-1b**	**B**	**B**	**B**	**6.5**	**2.6**	**10.5**	**1.62**	**0**
Mushk Budji	**A**	**A**	**A**	**5.2**	**1.6**	**7.7**	**1.48**	**2**

KLBC: Kernel Length Before Cooking (mm), LBR: Length Breadth Ratio, KLAC: Kernel Length After Cooking (mm), KER: Kernel Elongation Ratio, Aroma: (0-2 scale), A: Recipient parent allele, B: Donor Parent allele

Supplementary Table S3: Agronomic performance of two-gene BC_1F_1 derived BC_1F_2 plants of *Mushk Budji*/ DHMAS 70Q 164-1b

Plant ID	PH	NT	PL	SP	SF	GY
SKUA-485-9-1	115	7	15	193	93	8.4
SKUA-485-9-2	108	6	18	218	88	8.2
SKUA-485-9-3	105	5	20	210	80	6.6
SKUA-485-9-4	145	26	29	181	78	29.4
SKUA-485-9-5	143	28	18	222	89	38.9
SKUA-485-9-6	140	15	23	185	94	17.3
SKUA-485-12-1	121	12	22	183	89	13.7
SKUA-485-12-2	117	19	23	179	85	21.3
SKUA-485-12-3	113	17	24	175	92	18.6
SKUA-485-14-1	110	17	22	228	92	24.2
SKUA-485-14-2	115	22	27	218	81	30.0
SKUA-485-14-3	118	13	23	204	88	16.6
SKUA-485-17-1	110	12	22	212	94	15.9
SKUA-485-17-2	115	15	25	209	93	19.6
SKUA-485-17-3	120	12	22	195	85	14.6
SKUA-485-21-1	145	13	29	202	79	16.4
SKUA-485-21-2	118	14	25	177	82	15.5
SKUA-485-21-3	122	9	22	181	77	10.2
SKUA-485-22-1	110	11	22	223	78	15.3
SKUA-485-22-2	107	11	21	190	95	13.1
SKUA-485-22-3	115	18	21	180	91	20.3
SKUA-485-26-1	115	19	24	201	75	23.9
SKUA-485-26-2	122	9	21	218	77	12.3
SKUA-485-26-3	121	10	20	222	90	13.9

PH: Plant Height (cm), NT: Number of effective Tillers/ plant, PL: Panicle Length (cm), SP: Spikelets/ Plant, SF: Spikelet Fertility (%), GY: Grain Yield/ plant (g)

Supplementary Table S4: Kernel traits and cooking quality of two-gene BC_1F_1 derived BC_1F_2 plants of *Mushk Budji*/ DHMAS 70Q 164-1b

Plant ID	KLBC	LBR	KLAC	KER	Aroma	KLBC
SKUA-485-9-1	5.3	2.9	1.8	7.3	1.4	1
SKUA-485-9-2	5.0	3.0	1.7	7.0	1.4	1
SKUA-485-9-3	5.1	2.9	1.8	6.9	1.4	1
SKUA-485-9-4	5.1	3.0	1.7	7.2	1.4	1
SKUA-485-9-5	5.1	3.4	1.5	6.8	1.3	1
SKUA-485-9-6	4.9	3.0	1.6	7.3	1.5	1
SKUA-485-12-1	4.8	3.2	1.5	7.1	1.5	2
SKUA-485-12-2	5.2	3.4	1.5	6.8	1.3	2
SKUA-485-12-3	4.8	2.8	1.7	7.5	1.6	2
SKUA-485-14-1	5.0	3.5	1.4	6.8	1.4	1
SKUA-485-14-2	5.0	3.5	1.4	7.2	1.4	2
SKUA-485-14-3	4.9	3.5	1.4	7.5	1.5	1
SKUA-485-17-1	5.1	2.9	1.8	7.2	1.4	2
SKUA-485-17-2	5.0	2.9	1.7	7.3	1.5	2
SKUA-485-17-3	5.1	3.0	1.7	7.1	1.4	2
SKUA-485-21-1	4.8	3.1	1.5	7.4	1.5	2
SKUA-485-21-2	5.3	3.5	1.5	6.8	1.3	2
SKUA-485-21-3	4.8	3.5	1.4	7.5	1.6	2
SKUA-485-22-1	5.3	3.3	1.6	6.8	1.3	1
SKUA-485-22-2	4.8	3.3	1.5	7.5	1.6	1
SKUA-485-22-3	5.2	3.0	1.7	7.5	1.4	2
SKUA-485-26-1	4.9	3.4	1.4	7.2	1.5	1
SKUA-485-26-2	5.1	3.3	1.5	7.1	1.4	1
SKUA-485-26-3	4.9	3.0	1.6	6.9	1.4	1

KLBC: Kernel length before cooking (mm), KBBC: Kernel breadth before cooking (mm), LBR: Length breadth ratio, KLAC: Kernel length after cooking (mm), KER: Kernel elongation ratio, Aroma: (0-2 scale)

Supplementary Table S5: Agronomic performance of $BC_1F_{2:3}$ plants of *Mushk Budji*/ DHMAS 70Q 164-1b// *Mushk Budji*

Plant ID		PH	NT	PL	SP	SF	GY
**BC_1F_2**	**BC_1F_3**						
SKUA-485-27-13		120	20	23.0	95	68.4	11.9
	SKUA-485-27-13-1	117	24	24.5	142	82.4	21.3
	SKUA-485-27-13-2	126	18	27.0	143	93.7	16.1
	SKUA-485-27-13-3	127	10	23.0	172	91.3	30.8
	SKUA-485-27-13-4	129	14	27.5	157	89.2	13.7
	SKUA-485-27-13-5	127	13	22.5	139	84.2	11.3
	SKUA-485-27-13-6	118	10	24.0	153	91.5	29.6
	SKUA-485-27-13-7	130	13	23.5	149	85.9	12.1
	SKUA-485-27-13-8	129	14	23.0	153	92.2	13.4
	SKUA-485-27-13-9	125	18	25.0	137	84.7	15.4
	**Mean**	**125.33**	**14.89**	**24.44**	**149.44**	**88.33**	**18.81**
	**CV (%)**	**3.76**	**29.93**	**7.26**	**7.30**	**4.65**	**25.82**
SKUA-485-27-20		104	12	21	115	72.2	8.6
	SKUA-485-27-20-1	105	19	22.5	105	79.0	12.5
	SKUA-485-27-20-2	128	10	19.5	154	92.9	9.6
	SKUA-485-27-20-3	116	9	24.5	197	87.8	11.1
	SKUA-485-27-20-4	120	9	20.0	109	83.5	6.1
	SKUA-485-27-20-5	132	14	17.5	160	89.4	14.0
	SKUA-485-27-20-6	130	13	19.5	167	92.8	13.6
	SKUA-485-27-20-7	127	13	23.5	173	91.9	14.1
	SKUA-485-27-20-8	113	8	19.0	144	91.0	7.2
	SKUA-485-27-20-9	117	12	14.5	146	93.8	11.0
	SKUA-485-27-20-10	116	7	27.0	157	91.1	6.9
	SKUA-485-27-20-11	116	8	23.5	195	90.3	9.8
	SKUA-485-27-20-12	110	10	24.5	138	86.2	8.6
	SKUA-485-27-20-13	118	9	27.5	193	87.6	10.9
	**Mean**	**119.08**	**10.85**	**21.77**	**156.77**	**89.02**	**10.40**
	**CV (%)**	**6.77**	**30.31**	**17.48**	**18.75**	**4.72**	**25.85**
SKUA-485-27-32		115	15	23.0	152	88.8	14.3
	SKUA-485-27-32-1	129	12	25.5	184	96.2	23.8
	SKUA-485-27-32-2	127	6	20.5	139	89.2	15.2
	SKUA-485-27-32-3	130	13	26.5	186	89.8	15.1
	SKUA-485-27-32-4	115	11	22.5	160	96.9	11.0
	SKUA-485-27-32-5	123	12	27.5	150	94.0	21.3
	SKUA-485-27-32-6	116	12	24.0	109	75.2	8.2
	SKUA-485-27-32-7	123	9	23.0	105	72.4	5.9
	**Mean**	**123.29**	**10.71**	**24.21**	**147.57**	**87.67**	**10.07**
	**CV (%)**	**4.84**	**22.68**	**10.11**	**22.02**	**11.34**	**37.64**
SKUA-485-27-43		133	21	24	175	69.1	23.0
	SKUA-485-27-43-1	125	15	23.5	152	92.8	14.3
	SKUA-485-27-43-2	139	11	23.0	140	82.9	9.6
	SKUA-485-27-43-3	133	7	24.5	170	88.8	7.4
	SKUA-485-27-43-4	131	12	25.0	152	92.1	11.4
	SKUA-485-27-43-5	147	8	25.5	162	90.7	8.1
	SKUA-485-27-43-6	155	9	22.0	142	90.8	8.0
	SKUA-485-27-43-7	131	10	23.5	135	88.1	8.4
	SKUA-485-27-43-8	137	10	24.5	140	87.9	8.8
	SKUA-485-27-43-9	139	12	24.0	139	84.9	10.4
	**Mean**	**137.44**	**10.44**	**23.94**	**148.00**	**88.78**	**9.60**
	**CV (%)**	**6.62**	**23.01**	**4.48**	**8.02**	**3.70**	**22.44**
SKUA-485-27-47		118	13	22	92	89.1	7.5
	SKUA-485-27-47-1	134	17	23.0	135	91.1	14.3
	SKUA-485-27-47-2	133	15	24.0	143	90.2	13.4

	SKUA-485-27-47-3	130	22	24.5	147	91.2	20.2
	SKUA-485-27-47-4	139	18	27.5	193	90.2	21.7
	SKUA-485-27-47-5	138	17	26.5	194	88.1	20.6
	**Mean**	**134.80**	**17.80**	**25.10**	**162.40**	**90.16**	**18.06**
	**CV (%)**	**2.75**	**14.54**	**7.37**	**17.68**	**1.35**	**21.44**
SKUA-485-27-50		123	12	19	93	97.8	7.0
	SKUA-485-27-50-1	119	13	26.5	159	89.9	12.9
	SKUA-485-27-50-2	133	8	27.5	179	88.3	9.0
	SKUA-485-27-50-3	121	5	26.5	149	83.9	4.7
	SKUA-485-27-50-4	142	7	27.5	165	86.1	7.2
	SKUA-485-27-50-5	129	9	27.5	185	86.5	10.4
	SKUA-485-27-50-6	129	7	25.5	175	89.1	7.7
	**Mean**	**128.83**	**8.17**	**26.83**	**168.67**	**87.30**	**8.63**
	**CV (%)**	**6.48**	**33.23**	**3.04**	**7.99**	**2.57**	**32.91**
SKUA-485-27-56		132	11	22	100	89.0	6.9
	SKUA-485-27-56-1	157	7	24.5	115	83.5	5.0
	SKUA-485-27-56-2	153	18	25.5	109	86.2	12.3
	SKUA-485-27-56-3	145	17	27.0	197	87.8	20.9
	SKUA-485-27-56-4	140	18	26.5	105	86.7	11.8
	SKUA-485-27-56-5	134	12	22.0	103	87.4	7.7
	**Mean**	**145.80**	**14.40**	**25.10**	**125.80**	**86.32**	**11.55**
	**CV (%)**	**6.42**	**33.52**	**7.89**	**31.85**	**1.97**	**52.24**
SKUA-485-27-61		140	15	21	85	82.9	8.0
	SKUA-485-27-61-1	120	14	25.5	101	77.2	8.8
	SKUA-485-27-61-2	117	9	17.5	85	77.8	4.8
	SKUA-485-27-61-3	120	13	13.5	90	82.5	7.3
	SKUA-485-27-61-4	112	16	16.5	92	71.4	9.2
	SKUA-485-27-61-5	135	13	23.5	109	84.4	8.9
	SKUA-485-27-61-6	132	19	19.5	102	63.5	12.1
	**Mean**	**122.67**	**14.00**	**19.33**	**96.50**	**76.13**	**8.52**
	**CV (%)**	**7.29**	**23.90**	**23.23**	**9.29**	**10.10**	**28.29**
SKUA-485-27-64		135	12	23.0	174	64.9	13.1
	SKUA-485-27-64-1	139	20	27.0	65	84.6	8.1
	SKUA-485-27-64-2	156	17	24.5	85	82.4	9.0
	SKUA-485-27-64-3	158	9	25.5	75	84.0	4.2
	SKUA-485-27-64-4	145	14	21.5	112	83.0	9.8
	SKUA-485-27-64-5	148	9	26.5	77	88.3	4.3
	SKUA-485-27-64-6	141	10	27.0	105	93.3	6.6
	SKUA-485-27-64-7	137	18	23.5	153	85.6	17.2
	SKUA-485-27-64-8	134	20	23.0	146	87.0	18.3
	SKUA-485-27-64-9	144	10	21.5	135	88.1	8.4
	SKUA-485-27-64-10	141	7	28.0	117	90.6	5.1
	**Mean**	**144.30**	**13.40**	**24.80**	**107.00**	**86.70**	**9.11**
	**CV (%)**	**5.41**	**37.26**	**9.51**	**29.09**	**4.02**	**54.31**
SKUA-485-27-70		117	21	21	197	84.3	25.9
	SKUA-485-27-70-1	141	9	3.5	144	82.6	28.1
	SKUA-485-27-70-2	140	10	27.0	143	90.2	8.9
	SKUA-485-27-70-3	128	10	24.0	137	91.2	28.6
	SKUA-485-27-70-4	128	11	23.5	170	88.8	11.7
	SKUA-485-27-70-5	142	12	24.5	165	87.3	32.4
	**Mean**	**135.80**	**10.40**	**20.50**	**151.80**	**88.04**	**21.94**
	**CV (%)**	**5.27**	**10.96**	**46.82**	**9.68**	**3.82**	**19.67**
SKUA-485-27-77		127	20	21.0	122	49.2	15.3
	SKUA-485-27-77-1	145	17	23.5	109	91.7	11.6
	SKUA-485-27-77-2	132	21	22.0	107	89.7	24.0
	SKUA-485-27-77-3	123	19	23.0	111	90.2	18.2
	SKUA-485-27-77-4	114	22	22.5	105	93.3	24.4
	SKUA-485-27-77-5	117	18	24.0	102	85.3	15.5

	SKUA-485-27-77-6	136	20	25.5	101	94.1	18.6
	**Mean**	**127.83**	**19.50**	**23.42**	**105.83**	**90.73**	**18.72**
	**CV (%)**	**9.33**	**9.59**	**5.30**	**3.70**	**3.47**	**9.56**
SKUA-485-27-78-		**103**	**16**	**21.0**	**112**	**65.2**	**11.2**
	SKUA-485-27-78-1	125	18	22.0	105	88.6	11.8
	SKUA-485-27-78-2	117	21	23.5	112	84.8	14.7
	SKUA-485-27-78-3	132	19	22.5	97	80.4	11.5
	SKUA-485-27-78-4	114	17	19.0	77	81.8	8.2
	SKUA-485-27-78-5	126	18	19.0	45	71.1	5.1
	**Mean**	**122.80**	**18.60**	**21.20**	**87.20**	**81.35**	**10.26**
	**CV (%)**	**5.91**	**8.15**	**9.81**	**30.95**	**8.02**	**36.17**
SKUA-485-27-81-		120	17	21	111	81.4	11.8
	SKUA-485-27-81-1	117	9	22.5	133	94.7	7.5
	SKUA-485-27-81-2	127	6	24.0	129	92.2	4.8
	SKUA-485-27-81-3	124	7	19.5	143	91.6	6.3
	SKUA-485-27-81-4	136	4	23.5	`137	89.2	7.2
	SKUA-485-27-81-5	135	10	21.5	137	90.5	8.6
	SKUA-485-27-81-6	115	8	22.0	135	93.3	6.8
	SKUA-485-27-81-7	121	9	21.0	143	92.3	8.0
	SKUA-485-27-81-8	137	25	22.5	153	94.1	23.9
	**Mean**	**126.50**	**9.75**	**22.06**	**139.00**	**92.26**	**9.13**
	**CV (%)**	**6.89**	**66.19**	**6.46**	**5.76**	**1.99**	**66.58**
SKUA-485-27-86-		113	20	23.0	146	80.1	18.3
	SKUA-485-27-86-1	138	10	22.5	139	85.5	18.7
	SKUA-485-27-86-2	130	11	24.5	167	85.6	21.5
	SKUA-485-27-86-3	123	6	20.0	124	79.6	14.7
	SKUA-485-27-86-4	128	8	25.0	118	64.6	15.9
	SKUA-485-27-86-5	120	8	17.5	112	61.9	15.6
	SKUA-485-27-86-6	114	10	21.0	143	74.0	28.9
	SKUA-485-27-86-7	116	7	20.5	125	58.2	5.5
	SKUA-485-27-86-8	105	16	19.5	115	75.6	30.5
	SKUA-485-27-86-9	130	8	23.5	162	71.7	18.1
	SKUA-485-27-86-10	130	8	23.5	135	72.3	216.8
	**Mean**	**123.40**	**9.20**	**21.75**	**134.00**	**72.89**	**18.71**
	**CV (%)**	**7.91**	**30.66**	**11.17**	**14.21**	**12.78**	**31.85**
**DHMAS 70Q 164-2b**		**104**	**13**	**15**	**108**	**61**	**8.78**
Mushk Budji		**123**	**19**	**19**	**121**	**84**	**14.37**
	**Overall Mean**	**129.85**	**13.01**	**23.18**	**134.28**	**86.12**	**10.68**
	**Grand CV (%)**	**6.12**	**26.71**	**12.14**	**15.45**	**5.32**	**33.20**

PH: Plant Height (cm), NT: Number of effective Tillers/ plant, PL: Panicle Length (cm), SP: Spikelets/ Plant, SF: Spikelet Fertility (%), GY: Grain Yield/ plant (g)

Supplementary Table S6: Kernel traits and cooking quality of $BC_1F_{2:3}$ plants of *Mushk Budji*/DHMAS 70Q 164-1b//*Mushk Budji*

Plant ID		KLBC	LBR	KLAC	KER	Aroma
**BC_1F_2**	**BC_1F_3**					
SKUA-485-27-13		4.5	1.8	7.0	1.6	2
	SKUA-485-27-13-1	4.8	1.7	6.7	1.4	2
	SKUA-485-27-13-2	4.3	1.5	6.0	1.4	0
	SKUA-485-27-13-3	4.8	1.7	6.5	1.4	1
	SKUA-485-27-13-4	5.0	1.7	7.0	1.4	2
	SKUA-485-27-13-5	4.8	1.7	6.8	1.4	1
	SKUA-485-27-13-6	4.5	1.6	6.8	1.5	2
	SKUA-485-27-13-7	4.5	2.3	6.6	1.5	2
	SKUA-485-27-13-8	5.1	2.6	7.7	1.5	0
	SKUA-485-27-13-9	5.1	2.6	7.4	1.4	0
	**Mean**	**4.75**	**1.93**	**6.83**	**1.44**	
	**CV (%)**	**6.32**	**21.19**	**7.28**	**3.30**	
SKUA-485-27-20		5.0	2.5	8.2	1.64	1
	SKUA-485-27-20-1	4.8	1.6	6.1	1.3	0
	SKUA-485-27-20-2	5.0	1.7	6.0	1.2	2
	SKUA-485-27-20-3	5.1	1.7	6.6	1.3	1
	SKUA-485-27-20-4	4.3	1.6	5.9	1.4	0
	SKUA-485-27-20-5	4.8	1.7	6.5	1.4	2
	SKUA-485-27-20-6	5.1	2.0	7.0	1.4	0
	SKUA-485-27-20-7	5.8	1.9	7.0	1.2	1
	SKUA-485-27-20-8	5.1	1.8	6.7	1.3	2
	SKUA-485-27-20-9	5.1	2.0	6.7	1.3	1
	SKUA-485-27-20-10	5.1	1.7	6.9	1.3	1
	SKUA-485-27-20-11	5.4	2.2	7.6	1.4	2
	SKUA-485-27-20-12	5.1	1.7	6.9	1.3	0
	SKUA-485-27-20-13	5.4	1.8	7.2	1.3	2
	**Mean**	**5.08**	**1.79**	**6.70**	**1.32**	
	**CV (%)**	**7.07**	**9.01**	**7.29**	**4.70**	
SKUA-485-27-32		4.5	1.8	7.0	1.6	2
	SKUA-485-27-32-1	5.1	2.1	7.0	1.4	2
	SKUA-485-27-32-2	5.0	2.0	6.8	1.4	1
	SKUA-485-27-32-3	5.1	2.4	7.2	1.4	1
	SKUA-485-27-32-4	4.8	2.2	6.6	1.4	1
	SKUA-485-27-32-5	4.8	1.9	6.8	1.4	2
	SKUA-485-27-32-6	5.1	2.1	7.1	1.4	2
	SKUA-485-27-32-7	4.8	1.9	6.4	1.3	2
	**Mean**	**4.95**	**2.08**	**6.84**	**1.38**	
	**CV (%)**	**3.82**	**8.94**	**4.12**	**2.08**	
SKUA-485-27-43		5.0	2.0	7.6	1.52	0
	SKUA-485-27-43-1	5.8	2.9	7.2	1.3	0
	SKUA-485-27-43-2	5.1	2.3	6.8	1.3	0
	SKUA-485-27-43-3	5.8	2.0	7.0	1.2	0
	SKUA-485-27-43-4	5.1	2.0	7.0	1.4	0
	SKUA-485-27-43-5	5.1	1.7	6.8	1.3	0
	SKUA-485-27-43-6	5.1	2.1	6.8	1.3	0
	SKUA-485-27-43-7	5.4	2.2	6.8	1.3	0
	SKUA-485-27-43-8	5.1	1.7	7.2	1.4	0
	SKUA-485-27-43-9	5.0	1.8	6.8	1.4	0
	**Mean**	**5.28**	**2.06**	**6.93**	**1.32**	
	**CV (%)**	**5.40**	**17.51**	**2.50**	**4.58**	
SKUA-485-27-47		4.5	1.8	7.0	1.56	1
	SKUA-485-27-47-1	4.6	1.7	7.2	1.6	2
	SKUA-485-27-47-2	4.8	1.9	6.9	1.5	2

	SKUA-485-27-47-3	4.5	1.7	6.5	1.4	2
	SKUA-485-27-47-4	5.8	1.9	7.0	1.2	2
	SKUA-485-27-47-5	4.5	1.8	6.2	1.4	2
	**Mean**	**4.83**	**1.80**	**6.76**	**1.41**	
	**CV (%)**	**10.93**	**5.88**	**5.97**	**8.88**	
SKUA-485-27-50		4.5	1.8	7.0	1.56	1
	SKUA-485-27-50-1	5.3	2.6	6.7	1.3	0
	SKUA-485-27-50-2	5.5	2.8	7.2	1.3	2
	SKUA-485-27-50-3	5.8	2.3	7.3	1.3	0
	SKUA-485-27-50-4	5.5	2.0	7.1	1.3	1
	SKUA-485-27-50-5	5.3	2.0	6.9	1.3	0
	SKUA-485-27-50-6	5.0	1.8	6.8	1.4	2
	**Mean**	**5.38**	**2.25**	**7.00**	**1.30**	
	**CV (%)**	**4.88**	**16.69**	**3.38**	**2.52**	
SKUA-485-27-56		5.0	2.5	7.8	1.56	1
	SKUA-485-27-56-1	4.8	1.7	6.9	1.5	0
	SKUA-485-27-56-2	4.8	1.7	6.6	1.4	0
	SKUA-485-27-56-3	4.3	1.7	6.8	1.6	1
	SKUA-485-27-56-4	4.5	2.0	6.6	1.5	0
	**Mean**	**4.56**	**1.79**	**6.73**	**1.48**	
	**CV (%)**	**5.25**	**7.91**	**2.23**	**5.99**	
SKUA-485-27-64		4.4	1.8	7.0	1.6	1
	SKUA-485-27-64-1	5.1	2.6	7.5	1.5	1
	SKUA-485-27-64-2	5.0	2.0	6.7	1.3	2
	SKUA-485-27-64-3	5.3	1.9	7.2	1.4	1
	SKUA-485-27-64-4	5.1	2.3	7.3	1.4	1
	SKUA-485-27-64-5	5.3	2.6	7.2	1.4	1
	SKUA-485-27-64-6	4.8	1.9	6.9	1.5	2
	SKUA-485-27-64-7	5.1	2.1	7.0	1.4	1
	SKUA-485-27-64-8	5.0	2.0	6.9	1.4	1
	SKUA-485-27-64-9	5.8	2.9	7.6	1.3	2
	SKUA-485-27-64-10	5.8	2.3	7.4	1.3	1
	**Mean**	**5.21**	**2.25**	**7.17**	**1.38**	
	**CV (%)**	**6.09**	**15.08**	**4.06**	**4.06**	
SKUA-485-27-70		5.0	2.1	7.0	1.40	1
	SKUA-485-27-70-1	5.0	2.0	7.0	1.4	0
	SKUA-485-27-70-2	5.4	2.4	7.0	1.3	0
	SKUA-485-27-70-3	4.5	2.3	6.8	1.5	0
	SKUA-485-27-70-4	5.1	2.1	7.0	1.4	0
	SKUA-485-27-70-5	4.8	2.1	6.5	1.4	0
	**Mean**	**4.95**	**2.16**	**6.86**	**1.39**	
	**CV (%)**	**6.82**	**7.34**	**3.19**	**5.52**	
SKUA-485-27-77		4.5	2.3	8.4	1.9	1
	SKUA-485-27-77-1	4.8	1.9	6.7	1.4	1
	SKUA-485-27-77-2	5.4	2.2	7.2	1.3	0
	SKUA-485-27-77-3	5.3	2.1	7.2	1.4	0
	SKUA-485-27-77-4	5.3	2.1	7.2	1.4	2
	SKUA-485-27-77-5	5.0	2.0	6.7	1.3	1
	SKUA-485-27-77-6	5.4	2.4	7.1	1.3	0
	**Mean**	**5.17**	**2.11**	**7.02**	**1.36**	
	**CV (%)**	**4.76**	**7.82**	**3.54**	**2.36**	
SKUA-485-27-78		4.5	1.8	7.0	1.6	1
	SKUA-485-27-78-1	4.5	1.6	6.6	1.5	1
	SKUA-485-27-78-2	4.8	1.9	6.9	1.5	0
	SKUA-485-27-78-3	4.5	1.6	6.8	1.5	0
	SKUA-485-27-78-4	4.5	2.0	6.7	1.5	1
	SKUA-485-27-78-5	4.8	2.4	6.8	1.4	0
	**Mean**	**4.60**	**1.91**	**6.76**	**1.47**	

	**CV (%)**	**2.98**	**16.02**	**1.69**	**2.11**	
SKUA-485-27-81-		4.5	1.9	7.0	1.56	1
	SKUA-485-27-81-1	4.5	1.6	6.8	1.5	0
	SKUA-485-27-81-2	5.3	1.9	7.1	1.4	1
	SKUA-485-27-81-3	4.5	1.6	6.4	1.4	1
	SKUA-485-27-81-4	5.0	2.0	7.1	1.4	1
	SKUA-485-27-81-5	4.8	2.1	6.6	1.4	2
	SKUA-485-27-81-6	4.8	1.7	6.8	1.4	1
	SKUA-485-27-81-7	5.0	2.0	7.0	1.4	2
	SKUA-485-27-81-8	5.0	2.0	6.4	1.3	2
	**Mean**	**4.84**	**1.88**	**6.78**	**1.40**	
	**CV (%)**	**5.47**	**9.85**	**4.23**	**4.75**	
SKUA-485-27-86-		5.0	2.0	8.6	1.7	1
	SKUA-485-27-86-1	4.8	1.7	7.3	1.5	0
	SKUA-485-27-86-2	5.0	2.0	7.2	1.4	1
	SKUA-485-27-86-3	5.0	1.8	6.7	1.3	0
	SKUA-485-27-86-4	5.0	2.2	6.9	1.4	0
	SKUA-485-27-86-5	5.0	2.5	6.9	1.4	2
	SKUA-485-27-86-6	5.0	1.8	7.1	1.4	2
	SKUA-485-27-86-7	4.8	1.9	6.8	1.4	0
	SKUA-485-27-86-8	4.8	1.9	6.4	1.3	2
	SKUA-485-27-86-9	4.8	1.7	6.8	1.4	1
	SKUA-485-27-86-10	5.0	1.7	6.8	1.4	0
	**Mean**	**4.90**	**1.94**	**6.89**	**1.41**	
	**CV (%)**	**2.63**	**12.89**	**3.78**	**4.17**	
**DHMAS 70Q 164-1b**		**6.5**	**2.5**	**10.3**	**1.58**	**0**
Mushk Budji		**5.1**	**1.6**	**7.5**	**1.47**	**2**
	**Overall Mean**	**4.96**	**2.00**	**6.87**	**1.39**	
	**Grand CV (%)**	**5.64**	**11.62**	**4.00**	**4.22**	

KLBC: Kernel Length Before Cooking (mm), LBR: Length Breadth Ratio, KLAC: Kernel Length After Cooking (mm), KER: Kernel Elongation Ratio, Aroma: (0-2 scale)

Supplementary Table S7: Agronomic performance of BC_2F_2 plants of *Mushk Budji*/ DHMAS 70Q 164-1b// *Mushk Budji*// *Mushk Budji*

Plant ID	PH	NT	PL	SP	SF	GY	Decision
SKUA-485-27-4-							
SKUA-485-27-4-1	106	15	24.0	204	82.4	23.0	Selected
SKUA-485-27-4-2	118	15	23.5	215	87.4	24.1	Selected
SKUA-485-27-4-3	113	19	24.0	68	68.9	9.6	
SKUA-485-27-4-4	100	9	22.0	138	87.0	9.3	
SKUA-485-27-4-5	120	10	18.5	164	88.1	12.3	
SKUA-485-27-4-6	106	23	24.5	293	87.4	50.5	Selected
SKUA-485-27-4-7	125	4	23.5	221	87.1	6.6	
SKUA-485-27-4-8	130	8	17.5	138	91.3	8.3	
SKUA-485-27-4-9	100	14	25.5	164	78.9	17.2	
SKUA-485-27-4-10	115	12	24.0	143	74.7	12.8	Selected
SKUA-485-27-4-11	117	5	22.0	159	91.5	6.0	
SKUA-485-27-4-12	119	9	28.0	230	94.1	15.5	
SKUA-485-27-4-13	124	11	-	-	-	-	
SKUA-485-27-4-14	110	13	22.5	161	77.1	15.6	Selected
SKUA-485-27-4-15	122	17	17.7	71	59.6	9.0	Selected
SKUA-485-27-4-16	121	11	20.0	128	75.3	10.5	
SKUA-485-27-4-17	105	10	24.5	158	88.6	11.8	
SKUA-485-27-4-18	133	27	22.5	132	85.2	26.7	
SKUA-485-27-4-19	114	13	16.5	69	60.9	6.7	Selected
SKUA-485-27-4-20	107	7	20.8	128	60.0	6.7	
SKUA-485-27-4-21	97	7	25.0	159	89.6	8.3	
SKUA-485-27-4-22	99	7	27.5	168	78.6	8.8	
SKUA-485-27-4-23	115	7	21.5	200	85.0	10.5	Selected
SKUA-485-27-4-24	106	7	27.5	203	85.9	10.6	Selected
SKUA-485-27-4-25	113	7	22.5	158	91.4	8.3	
SKUA-485-27-4-26	104	10	26.0	221	84.4	16.5	
SKUA-485-27-4-27	126	7	21.5	146	76.3	7.6	Selected
SKUA-485-27-4-28	124	10	22.5	278	96.2	20.8	
SKUA-485-27-4-29	117	7	22.5	167	81.1	8.7	
SKUA-485-27-4-30	97	9	27.5	230	96.1	15.5	
SKUA-485-27-4-31	113	8	20.5	86	87.7	5.1	
SKUA-485-27-4-32	118	8	27.0	203	85.2	12.2	Selected
SKUA-485-27-4-33	131	11	23.5	158	78.6	13.0	Selected
SKUA-485-27-4-34	116	11	17.5	213	86.6	17.6	
SKUA-485-27-4-35	125	11	23.5	221	89.8	18.2	
SKUA-485-27-4-36	121	8	16.5	203	92.6	12.2	
SKUA-485-27-4-37	129	10	19.5	146	85.6	10.9	
SKUA-485-27-4-38	123	16	19.0	80	82.3	9.5	Selected
SKUA-485-27-4-39	107	4	13.7	71	76.6	2.1	
SKUA-485-27-4-40	114	9	17.5	65	80.0	7.4	Selected
SKUA-485-27-4-41	127	5	20.0	105	87.1	3.9	
SKUA-485-27-4-42	126	4	18.5	101	79.1	3.0	
SKUA-485-27-4-43	112	4	19.5	74	63.3	2.2	
SKUA-485-27-4-44	130	5	20.5	134	88.8	5.0	
SKUA-485-27-4-45	129	5	23.5	276	92.9	10.4	
SKUA-485-27-4-46	110	6	24.5	269	87.2	12.1	
SKUA-485-27-4-47	114	10	25.5	278	84.1	20.8	
SKUA-485-27-4-48	112	12	19.0	293	96.9	26.3	

SKUA-485-27-4-49	113	5	23.5	179	94.1	6.7	
SKUA-485-27-4-50	107	6	24.5	158	77.1	7.1	Selected
SKUA-485-27-4-51	122	9	20.0	161	86.9	10.8	
SKUA-485-27-4-52	122	9	20.5	171	82.5	11.5	Selected
SKUA-485-27-4-53	95	6	14.5	164	86.2	7.4	
SKUA-485-27-4-54	127	7	24.5	159	82.1	8.3	Selected
SKUA-485-27-4-55	103	9	19.5	168	83.0	11.3	
SKUA-485-27-4-56	122	7	20.0	131	88.5	6.9	
SKUA-485-27-4-57	120	15	15.5	218	83.4	24.5	Selected
SKUA-485-27-4-58	137	10	22.0	203	81.5	15.2	Selected
SKUA-485-27-4-59	105	5	22.5	143	82.1	5.3	
SKUA-485-27-4-60	103	5	24.0	221	87.1	8.3	
SKUA-485-27-4-61	121	6	20.5	132	77.3	5.9	Selected
SKUA-485-27-4-62	121	4	20.5	98	81.5	2.9	
SKUA-485-27-4-63	123	7	16.6	68	66.7	3.5	
SKUA-485-27-4-64	110	4	20.0	221	93.9	6.6	
SKUA-485-27-4-65	128	4	25.5	288	90.1	8.6	
SKUA-485-27-4-66	129	4	22.2	165	90.0	5.0	
SKUA-485-27-4-67	124	4	24.5	296	92.4	8.9	
SKUA-485-27-4-68	116	6	23.5	293	96.4	13.2	
SKUA-485-27-4-69	104	7	23.5	218	91.0	11.4	
SKUA-485-27-4-70	132	3	21.0	206	92.0	4.6	
SKUA-485-27-4-71	107	4	26.5	170	91.2	5.1	
SKUA-485-27-4-72	106	5	23.5	231	92.9	8.7	
SKUA-485-27-4-73	109	6	19.5	158	81.0	7.1	
SKUA-485-27-4-74	97	3	20.5	116	84.4	2.6	
SKUA-485-27-4-75	106	7	17.5	99	78.8	5.2	
SKUA-485-27-4-76	132	5	19.5	110	67.1	4.1	
SKUA-485-27-4-77	107	7	15.0	80	81.1	4.2	
SKUA-485-27-4-78	115	10	24.5	168	80.4	12.6	
SKUA-485-27-4-79	158	13	19.5	83	60.0	8.0	Selected
SKUA-485-27-4-80	136	5	23.5	168	81.3	6.3	
SKUA-485-27-4-81	121	9	26.0	233	91.0	15.7	
SKUA-485-27-4-82	120	6	14.5	146	88.7	6.5	
SKUA-485-27-4-83	122	9	19.0	231	70.8	15.6	
SKUA-485-27-4-84	130	5	22.5	296	93.4	11.1	
SKUA-485-27-4-85	132	12	21.5	285	94.2	25.7	
SKUA-485-27-4-86	119	6	20.5	180	90.8	8.1	
SKUA-485-27-4-87	110	6	109.0	155	79.6	7.0	Selected
SKUA-485-27-4-88	134	7	17.5	161	82.2	8.4	
SKUA-485-27-4-89	107	7	19.5	168	83.9	8.8	
SKUA-485-27-4-90	114	5	15.0	137	73.6	5.1	
SKUA-485-27-4-91	121	8	24.5	128	83.5	7.7	
SKUA-485-27-4-92	110	5	19.5	171	79.8	6.4	
SKUA-485-27-4-93	121	7	23.5	128	75.3	6.7	
SKUA-485-27-4-94	120	9	26.0	174	88.8	11.7	
SKUA-485-27-4-95	109	7	14.5	132	85.2	6.9	
SKUA-485-27-4-96	149	9	19.0	170	89.4	11.4	
SKUA-485-27-4-97	125	9	22.5	153	87.3	10.3	
SKUA-485-27-4-98	117	18	21.5	176	90.6	23.7	
**Family mean**	**117.22 ±1.92**	**8.45 ±0.73**	**22.36 ±1.66**	**170 ±9.82**	**83.43 ±1.89**	**10.77 ±1.16**	
**CV (%)**	**9.68**	**49.67**	**42.46**	**35**	**10.67**	**65.40**	

SKUA-485-27-7-							
SKUA-485-27-7-1	113	13	17.5	170	81.4	16.5	
SKUA-485-27-7-2	125	15	14.0	198	86.4	22.3	
SKUA-485-27-7-3	134	13	27.0	195	90.0	19.0	Selected
SKUA-485-27-7-4	132	14	27.0	218	82.1	22.8	
SKUA-485-27-7-5	126	10	27.0	180	80.8	13.5	Selected
SKUA-485-27-7-6	114	13	15.7	218	85.5	21.2	
SKUA-485-27-7-7	125	16	15.0	105	74.3	12.6	
SKUA-485-27-7-8	127	10	18.5	90	66.7	6.8	Selected
SKUA-485-27-7-9	129	11	45.0	240	93.8	19.8	Selected
SKUA-485-27-7-10	114	15	16.5	200	84.2	22.4	
SKUA-485-27-7-11	125	14	45.0	231	93.5	24.3	Selected
SKUA-485-27-7-12	121	14	16.3	168	77.7	17.6	Selected
SKUA-485-27-7-13	114	13	15.7	218	85.5	21.2	Selected
SKUA-485-27-7-14	125	16	15.0	105	74.3	12.6	Selected
SKUA-485-27-7-15	121	11	17.5	158	77.1	13.0	Selected
SKUA-485-27-7-16	114	8	18.5	81	81.5	4.9	
SKUA-485-27-7-17	114	12	22.0	120	77.5	10.8	Selected
SKUA-485-27-7-18	112	16	27.5	288	89.1	34.6	
SKUA-485-27-7-19	143	13	22.5	173	92.2	16.8	
SKUA-485-27-7-20	112	18	27.5	171	81.6	23.1	
SKUA-485-27-7-21	143	12	23.5	173	86.1	15.5	
SKUA-485-27-7-22	114	13	24.0	81	77.8	7.9	
SKUA-485-27-7-23	154	9	25.5	264	85.5	17.8	
SKUA-485-27-7-24	104	9	22.5	203	88.9	13.7	
SKUA-485-27-7-25	135	8	20.0	83	60.0	5.0	Selected
SKUA-485-27-7-26	114	9	18.0	81	77.8	5.5	Selected
SKUA-485-27-7-27	154	10	25.5	270	86.1	20.3	
SKUA-485-27-7-28	104	9	22.5	203	91.9	13.7	
SKUA-485-27-7-29	97	6	23.5	249	86.1	11.2	
SKUA-485-27-7-30	123	11	25.6	231	85.1	19.1	
SKUA-485-27-7-31	133	6	13.0	96	67.2	4.3	Selected
SKUA-485-27-7-32	104	9	22.5	203	91.9	13.7	
SKUA-485-27-7-33	123	10	21.5	180	89.2	13.5	Selected
SKUA-485-27-7-34	125	17	16.0	105	74.3	13.4	
SKUA-485-27-7-35	144	8	26.5	270	86.7	16.2	Selected
SKUA-485-27-7-36	104	9	35.0	165	89.1	11.1	Selected
SKUA-485-27-7-37	104	14	28.5	248	85.5	26.0	
SKUA-485-27-7-38	117	12	21.5	120	76.3	10.8	Selected
SKUA-485-27-7-39	112	16	27.5	288	89.1	34.6	
SKUA-485-27-7-40	143	13	22.5	173	92.2	16.8	
**Family mean**	**122.28 ±2.31**	**11.88 ±0.58**	**22.90 ±1.21**	**180 ±10.87**	**83.04 ±1.18**	**16.14 ±2.57**	
**CV (%)**	**11.43**	**25.53**	**31.10**	**35**	**9.34**	**43.92**	
SKUA-485-27-8-							
SKUA-485-27-8-1	123	12	25	213	91.5	19.2	Selected
SKUA-485-27-8-2	116	10	23	185	82.1	13.8	
SKUA-485-27-8-3	114	9	18	81	77.8	15.5	Selected
SKUA-485-27-8-4	145	13	19	158	78.1	15.4	
SKUA-485-27-8-5	156	16	22.5	158	91.4	18.9	Selected
SKUA-485-27-8-6	104	13	27.5	263	85.7	25.6	
SKUA-485-27-8-7	154	10	25.5	270	86.1	20.3	Selected
SKUA-485-27-8-8	104	9	22.5	203	91.9	13.7	Selected

SKUA-485-27-8-9	97	6	23.5	249	86.1	11.2	Selected
SKUA-485-27-8-10	100	7	21	176	81.2	9.2	
SKUA-485-27-8-11	108	13	18.5	116	87.0	11.3	Selected
SKUA-485-27-8-12	134	18	15.5	99	77.3	13.4	Selected
SKUA-485-27-8-13	144	23	21.3	218	82.1	37.5	Selected
SKUA-485-27-8-14	104	22	27	221	88.4	36.4	
SKUA-485-27-8-15	117	13	19	98	58.5	9.5	
SKUA-485-27-8-16	121	14	25.5	203	85.9	21.3	
SKUA-485-27-8-17	116	13	27.5	198	90.9	19.3	
SKUA-485-27-8-18	125	16	25	161	87.9	19.3	
SKUA-485-27-8-19	121	11	25.5	233	86.5	19.2	
SKUA-485-27-8-20	108	13	25.5	239	94.3	23.3	
SKUA-485-27-8-21	135	8	28	233	89.0	14.0	
SKUA-485-27-8-22	138	9	23	209	90.6	14.1	
SKUA-485-27-8-23	133	8	22.5	188	88.8	11.3	
SKUA-485-27-8-24	123	11	21.5	135	86.7	11.1	
SKUA-485-27-8-25	145	8	23.5	165	89.1	9.9	
SKUA-485-27-8-26	123	11	22.5	113	74.7	9.3	
SKUA-485-27-8-27	127	11	17	68	57.8	5.6	
SKUA-485-27-8-28	121	19	24.5	174	81.0	24.8	Selected
SKUA-485-27-8-29	128	19	22	158	82.9	22.4	
SKUA-485-27-8-30	122	12	21.5	233	88.4	20.9	
SKUA-485-27-8-31	117	15	22	249	91.0	28.0	
SKUA-485-27-8-32	124	14	20	203	88.1	21.3	
SKUA-485-27-8-33	122	17	19.5	182	88.4	23.1	
SKUA-485-27-8-34	107	12	19	171	89.5	15.4	
SKUA-485-27-8-35	133	14	27	215	85.3	22.5	
SKUA-485-27-8-36	137	7	28	239	85.5	12.5	
SKUA-485-27-8-37	133	8	26.5	198	83.3	11.9	
**Family mean**	**123.76 ±2.5**	**12.54 ±0.66**	**22.86 ±0.54**	**185 ±8.67**	**84.62 ±1.35**	**17.59 ±1.30**	
**CV (%)**	**11.84**	**33.02**	**14.42**	**28**	**9.27**	**43.02**	
**DHMAS 70Q 164-1b**	**104**	**13**	**15**	**108**	**61**	**8.78**	
Mushk Budji	**123**	**19**	**19**	**121**	**84**	**14.37**	

PH: Plant Height (cm), NT: Number of effective Tillers/ plant, PL: Panicle Length (cm), SP: Spikelets/ Plant, SF: Spikelet Fertility (%), GY: Grain Yield/ plant (g)

Supplementary Table S8: Marker-assisted foreground selection, evaluation for kernel traits and cooking quality of selected BC_2F_2 progenies

Plant ID	KLBC	KBBC	LBR	KLAC	KER	Aroma	Gene status		
SKUA-485-27-4-							***Pita***	***Pi1***	***Pi54***
SKUA-485-27-4-1	5.0	3.3	1.5	8.7	1.7	2	B	H	-
SKUA-485-27-4-2	5.3	3.2	1.7	7.2	1.3	1	B	H	-
SKUA-485-27-4-6	5.0	2.3	2.1	8.2	1.6	1	B	H	-
SKUA-485-27-4-10	4.5	2.7	1.7	8.0	1.8	1	B	H	-
SKUA-485-27-4-14	5.0	2.7	1.9	8.7	1.7	0	B	H	-
SKUA-485-27-4-15	5.0	3.5	1.4	7.3	1.5	0	A	B	-
SKUA-485-27-4-19	5.5	3.2	1.7	8.3	1.5	2	B	H	-
SKUA-485-27-4-23	5.5	3.5	1.6	7.8	1.4	2	B	-	-
SKUA-485-27-4-24	5.0	3.2	1.6	8.2	1.6	1	A	-	-
SKUA-485-27-4-27	4.5	3.5	1.3	7.7	1.7	1	A	A	-
SKUA-485-27-4-32	5.5	3.2	1.7	8.0	1.5	2	B	H	-
SKUA-485-27-4-33	5.5	3.3	1.7	7.5	1.4	1	A	-	-
SKUA-485-27-4-38	4.5	3.3	1.4	8.0	1.8	2	B	B	B
SKUA-485-27-4-40	5.5	3.3	1.7	8.2	1.5	2	B	B	B
SKUA-485-27-4-50	5.5	3.3	1.7	8.2	1.5	2	A	A	-
SKUA-485-27-4-52	5.0	3.5	1.4	8.7	1.7	0	B	A	-
SKUA-485-27-4-54	5.5	3.5	1.6	8.2	1.5	0	A	-	-
SKUA-485-27-4-57	5.5	3.2	1.7	7.7	1.4	0	A	B	-
SKUA-485-27-4-58	5.5	3.3	1.7	7.9	1.4	1	A	B	-
SKUA-485-27-4-61	5.5	3.0	1.8	8.2	1.5	2	A	H	-
SKUA-485-27-4-79	5.5	3.2	1.7	8.0	1.5	1	A	H	-
SKUA-485-27-4-87	5.5	3.3	1.7	7.5	1.4	1	A	H	-
**Mean**	**5.22 ±0.07**	**3.20 ±0.05**	**1.64 ±0.03**	**8.00 ±0.08**	**1.54 ±0.03**				
**CV (%)**	**6.99**	**9.49**	**11.27**	**5.11**	**9.47**				

Plant ID	KLBC	KBBC	LBR	KLAC	KER	Aroma	Gene status		
SKUA-485-27-7-							***Pita***	***Pi1***	***Pi54***
SKUA-485-27-7-3	5.0	3.0	1.7	8.2	1.6	2	A	B	-
SKUA-485-27-7-5	4.8	2.0	2.4	9.2	1.9	1	A	A	-
SKUA-485-27-7-8	5.3	2.2	2.5	7.7	1.4	2	A	H	-
SKUA-485-27-7-9	4.2	2.2	1.9	7.3	1.8	1	A	B	-
SKUA-485-27-7-11	5.2	2.2	2.4	7.2	1.4	1	A	A	-
SKUA-485-27-7-12	5.0	2.5	2.0	8.2	1.6	1	A	H	-
SKUA-485-27-7-13	5.0	3.0	1.7	7.3	1.5	2	B	H	-
SKUA-485-27-7-14	4.5	2.0	2.3	7.2	1.6	1	A	A	-
SKUA-485-27-7-15	5.0	2.5	2.0	7.8	1.6	2	B	H	-
SKUA-485-27-7-17	5.5	3.0	1.8	9.2	1.7	1	B	H	-
SKUA-485-27-7-25	5.0	3.0	1.7	7.7	1.5	2	A	H	-
SKUA-485-27-7-26	5.3	3.0	1.8	8.5	1.6	2	B	A	-
SKUA-485-27-7-31	5.0	2.5	2.0	7.5	1.5	2	A	H	-
SKUA-485-27-7-33	5.0	3.0	1.7	7.8	1.6	2	A	H	-
SKUA-485-27-7-35	5.0	2.5	2.0	7.3	1.5	1	B	H	-
SKUA-485-27-7-36	5.0	3.0	1.7	7.5	1.5	2	A	A	-
SKUA-485-27-7-38	5.3	3.0	1.8	8.7	1.7	2	A	A	-
**Mean**	**5.00 ±0.06**	**2.62 ±0.10**	**1.95 ±0.07**	**7.89 ±0.16**	**1.58 ±0.03**				
**CV (%)**	**6.10**	**15.39**	**14.43**	**8.24**	**7.92**				

**Plant ID**	**KLBC**	**KBBC**	**LBR**	**KLAC**	**KER**	**Aroma**	**Gene status**		
SKUA-485-27-8-							***Pita***	***Pi1***	***Pi54***
SKUA-485-27-8-1	4.3	3.0	1.4	7.7	1.8	2	B	A	-
SKUA-485-27-8-3	5.0	2.5	2.0	8.3	1.7	1	B	B	B
SKUA-485-27-8-5	4.3	2.7	1.6	7.5	1.7	2	B	-	-
SKUA-485-27-8-7	4.5	2.2	2.1	7.7	1.7	1	B	A	-
SKUA-485-27-8-8	4.5	2.7	1.7	7.8	1.7	0	A	A	-
SKUA-485-27-8-9	4.7	2.7	1.8	8.3	1.8	1	B	A	-
SKUA-485-27-8-11	4.3	2.0	2.2	8.0	1.8	2	B	A	-
SKUA-485-27-8-12	5.0	3.2	1.6	8.7	1.7	0	A	A	-
SKUA-485-27-8-13	4.7	2.3	2.0	8.2	1.8	0	B	A	-
SKUA-485-27-8-28	4.7	3.0	1.6	7.7	1.6	1	A	A	-
**Mean**	**4.59 ±0.08**	**2.62 ±0.12**	**1.79 ±0.08**	**7.98 ±0.33**	**1.74 ±0.02**				
**CV (%)**	**5.70**	**14.42**	**14.34**	**4.77**	**3.52**				
**DHMAS 70Q 164-1b**	**6.5**	**2.6**	**2.5**	**10.3**	**1.58**	**0**			
Mushk Budji	**5.1**	**3.2**	**1.6**	**7.5**	**1.47**	**2**			

KLBC: Kernel Length Before Cooking (mm), KBBC: Kernel Breadth Before Cooking, LBR: Length Breadth Ratio, KLAC: Kernel Length After Cooking (mm), KER: Kernel Elongation Ratio, Aroma: (0-2 scale), A: Recipient parent allele, B: Donor Parent allele

Supplementary Table S9. Fold-change in protein expression under 2D gel electrophoresis of three-gene pyramided as compared to donor line DHMAS 70Q 164-1b

Protein Name	Match ID	Max	Fold	Match Count	Mushk Budji	SKUA-485-27-4-38-4	SKUA-485-27-4-40-6	DHMAS 70Q 164-1b	ANOVA
Alpha-amylase	33	0.75007	1.82084	4	0.75007	0.70812	0.74073	0.41193	0.00467
Triosephosphate isomerase	47	1.41549	16.53680	4	0.30501	0.30613	1.41549	0.08560	0.00009
19 kDa globulin	109	0.80443	8.06367	4	0.42705	0.29868	0.80443	0.09976	0.00249
S-(+)-linalool synthase	133	0.50076	1.81634	3	0.50076	0.33239	0.27570	-	0.00000

Supplementary Table S10. Grain yield performance of pyramided lines across different locations of Kashmir valley

S. No.	Line ID	Grain yield (t/ha) (GY)					
		E_1	E_2	E_3	E_4	E_5	Mean
1	SKUA-485-27-50-5-5	3.23	3.02	2.98	2.74	2.81	2.96±0.09
2	SKUA-485-27-20-6-4	5.13	4.68	4.12	3.74	3.03	4.14±0.36
3	SKUA-485-27-13-1-3	3.85	3.52	2.98	3.33	2.86	3.31±0.18
4	SKUA-485-27-86-10-4	5.95	4.93	4.63	4.38	4.40	4.86±0.29
5	SKUA-485-27-77-6-2	5.61	5.12	4.62	4.25	4.16	4.75±0.27
6	SKUA-485-27-47-4-1	4.21	5.34	5.21	4.61	4.82	4.84±0.20
7	SKUA-485-27-3-7-5	5.34	5.26	4.38	4.13	3.00	4.42±0.43
8	SKUA-485-27-4-38-4	5.51	5.56	5.24	4.20	3.33	4.77±0.44
9	SKUA-485-27-4-40-6	5.62	5.40	4.70	4.54	3.89	4.83±0.31
10	Mushk Budji	5.12	4.89	4.38	3.85	3.42	4.33±0.32
11	DHMAS 70Q 164-1b	1.62	1.85	2.11	2.58	2.45	2.12±0.18
	CD (P<0.05)	1.45	1.28	1.20	0.95	1.05	-
	Coefficient of variation (%)	8.89	8.28	5.85	6.50	7.53	-

Supplementary Table S11. Agronomic performance of pyramided lines for days to flowering and primary yield attributes under different locations within Kashmir valley

S. No.	Line ID	Number of effective tillers per plant (NT)						Spikelets per panicle (SP)					
		E_1	E_2	E_3	E_4	E_5	Mean	E_1	E_2	E_3	E_4	E_5	Mean
1	SKUA-485-27-50-5-5	10.25	11.12	11.56	10.84	10.12	10.78±0.27	131.30	113.16	107.41	101.11	115.69	113.73±5.06
2	SKUA-485-27-20-6-4	14.50	13.87	13.58	13.21	12.58	13.55±0.32	141.52	140.59	121.35	113.25	96.34	122.61±8.54
3	SKUA-485-27-13-1-3	12.50	13.14	12.85	12.34	11.86	12.54±0.22	128.33	107.15	92.76	112.44	100.48	108.23±6.01
4	SKUA-485-27-86-10-4	15.21	15.10	15.34	14.35	13.87	14.77±0.28	163.00	130.60	125.76	122.09	126.89	133.67±7.46
5	SKUA-485-27-77-6-2	14.96	14.57	14.15	13.24	13.56	14.10±0.32	156.25	146.42	130.60	133.75	122.71	137.95±5.96
6	SKUA-485-27-47-4-1	16.83	17.14	16.38	16.85	15.23	16.49±0.34	100.04	124.62	132.53	114.00	131.59	120.56±6.11
7	SKUA-485-27-3-7-5	14.00	14.23	14.28	13.85	13.69	14.01±0.11	158.93	147.86	127.80	124.25	91.31	130.03±11.60
8	SKUA-485-27-4-38-4	14.67	15.20	14.14	14.52	14.24	14.55±0.19	150.24	152.41	132.96	115.70	93.54	128.97±11.07
9	SKUA-485-27-4-40-6	15.24	14.95	13.85	14.64	13.23	14.38±0.37	147.51	150.50	151.34	124.04	122.51	139.18±6.53
10	Mushk Budji	14.18	14.34	13.26	12.85	12.50	13.43±0.36	144.43	136.40	132.13	119.84	114.00	129.36±5.53
11	DHMAS 70Q 164-1b	10.23	10.15	11.52	10.25	11.89	10.81±0.37	87.98	101.26	101.76	132.48	108.45	106.38±7.32
	CD (P<0.05)	2.49	2.17	1.84	2.24	1.98	-	25.97	22.15	22.20	12.38	20.83	-
	Coefficient of variation (%)	8.12	9.51	6.52	5.14	7.31	-	6.23	8.12	5.44	6.20	7.58	-

Contd..

S. No.	Line ID	1000-seed weight (SW)						Days to 50% flowering					
		**E_1**	**E_2**	**E_3**	**E_4**	**E_5**	**Mean**	**E_1**	**E_2**	**E_3**	**E_4**	**E_5**	**Mean**
1	SKUA-485-27-50-5-5	24.59	24.61	24.32	25.67	24.96	24.83±0.23	100.2	108.5	92.8	95.8	89.2	97.3±3.33
2	SKUA-485-27-20-6-4	25.41	24.88	25.37	25.60	25.60	25.37±0.13	95.3	97.2	80.6	83.4	80.3	87.4±3.68
3	SKUA-485-27-13-1-3	24.40	25.12	25.41	24.85	24.30	24.82±0.21	102.5	100.5	89.2	94.5	88.1	95.0±2.90
4	SKUA-485-27-86-10-4	24.14	25.58	24.25	25.79	25.41	25.03±0.35	96.5	93.4	81.6	85.7	84.1	88.3±2.85
5	SKUA-485-27-77-6-2	24.61	24.15	25.27	24.59	25.78	24.88±0.29	96.2	99.2	83.1	82.2	78.8	87.9±4.09
6	SKUA-485-27-47-4-1	25.21	25.41	24.65	24.63	24.78	24.94±0.16	93.1	99.1	81.4	84.7	80.4	87.7±3.61
7	SKUA-485-27-3-7-5	24.20	25.81	24.27	24.36	24.83	24.69±0.30	90.4	96.2	82.8	79.5	75.2	84.8±3.78
8	SKUA-485-27-4-38-4	25.44	24.95	25.82	25.49	25.63	25.47±0.14	93.5	97.0	85.1	83.6	79.4	87.7±3.26
9	SKUA-485-27-4-40-6	25.42	24.30	25.51	25.67	24.53	25.09±0.28	97.2	101.2	87.8	85.2	80.7	90.4±3.81
10	Mushk Budji	25.20	25.58	25.50	25.30	24.26	25.17±0.24	95.4	100.4	85.2	85.3	80.7	89.4±3.65
11	DHMAS 70Q 164-1b	18.59	18.91	18.32	19.11	19.60	18.91±0.22	120.3	125.5	115.8	113.8	110.1	117.1±2.67
	CD (P<0.05)	0.74	0.79	0.84	0.75	0.79	-	5.21	4.95	6.88	5.50	4.82	-
	Coefficient of variation (%)	3.12	4.64	2.58	2.90	3.33	-	3.82	5.65	4.48	6.30	7.21	-

Supplementary Table S12. Physico-chemical and cooking quality traits of pyramided lines

**Line ID**	**Plant ID**	**KLBC (mm)**	**LBR**	**KLAC (mm)**	**KER**	**GC**	**AC (%)**	**Hulling (%)**	**Milling (%)**	**ASV**	**Aroma (0-2 scale)**
1	SKUA-485-27-50-5-5	4.89±0.08	1.82±0.02	8.18±0.12	1.67±0.03	92±4.12	15.21±0.90	80.46±2.33	70.77±3.10	4	1
2	SKUA-485-27-20-6-4	5.34±0.07	1.77±0.03	8.21±0.11	1.54±0.02	95±5.55	18.12±1.12	84.54±3.14	74.32±3.67	4	2
3	SKUA-485-27-13-1-3	5.45±0.07	1.80.±0.04	8.41±0.09	1.54±0.02	96±6.28	17.45±0.85	83.15±1.87	73.15±3.15	4	2
4	SKUA-485-27-86-10-4	5.33±0.08	1.75±0.02	8.39±0.10	1.57±0.03	93±5.80	17.66±0.95	86.15±2.45	76.89±2.95	4	2
5	SKUA-485-27-77-6-2	5.52±0.10	1.75±0.02	8.43±0.12	1.53±0.04	98±4.15	19.35±1.02	74.62±3.66	74.42±3.11	4	2
6	SKUA-485-27-47-4-1	5.13±0.07	1.85±0.03	7.88±0.11	1.54±0.03	95±6.32	16.82±1.20	78.12±3.50	69.10±2.74	4	2
7	SKUA-485-27-3-7-5	5.11±0.08	1.72±0.02	8.31±0.10	1.63±0.02	93±3.19	17.71±0.82	86.24±2.94	78.12±3.68	4	2
8	SKUA-485-27-4-38-4	5.51±0.10	1.63±0.02	8.11±0.08	1.47±0.02	97±4.66	18.45±0.90	84.52±4.45	76.89±4.00	5	2
9	SKUA-485-27-4-40-6	5.5±0.09	1.70±0.04	8.26±0.10	1.50±0.03	97±5.87	18.91±0.94	85.61±3.77	75.32±3.45	5	2
10	*Mushk Budji*	5.45±0.08	1.65±0.03	8.13±0.11	1.49±0.02	115±6.30	14.14±1.05	86.25±4.66	78.25±3.10	4	2
11	DHMAS 70Q 164-1b	6.53±0.08	2.64±0.03	10.52±0.14	1.61±0.03	55±3.21	21.52±0.88	82.23±3.58	70.33±2.82	6	0
	CD (P<0.05)	0.14	0.05	0.11	0.04	4.42	1.09	2.81	2.05		
	Coefficient of variation (%)	3.12	2.45	3.34	2.56	2.66	4.18	5.64	5.28		

Supplementary Table S13. Disease reaction under uniform blast nursery across five hot spot locations

S. No.	Plant ID	Gene combination	Leaf blast reaction				
			Sagam	Pombay	Khudwani	Shalimar	Budgam
1	SKUA-485-27-50-5-5	*Pi54+Pi1+Pita*	5	3	4	5	2
2	SKUA-485-27-20-6-4	*Pi54+Pi1+Pita*	1	0	0	0	0
3	SKUA-485-27-13-1-3	*Pi54+Pi1+Pita*	3	1	2	0	2
4	SKUA-485-27-86-10-4	*Pi54+Pi1+Pita*	1	0	0	0	0
5	SKUA-485-27-77-6-2	*Pi54+Pi1+Pita*	1	0	0	0	1
6	SKUA-485-27-47-4-1	*Pi54+Pi1*	1	2	0	1	2
7	SKUA-485-27-3-7-5	*Pi54+Pi1+Pita*	1	0	0	0	0
8	SKUA-485-27-4-38-4	*Pi54+Pi1+Pita*	1	0	0	0	0
9	SKUA-485-27-4-40-6	*Pi54+Pi1+Pita*	1	1	0	1	0
10	Mushk Budji		9	5	8	7	8
11	DHMAS 70Q 164-1b	*Pi54+Pi1+Pita*	1	0	0	0	0

Field reaction scoring was carried out on 0-9 scale as per SES IRRI (1996); Score 0-3: R; 4-5: MR; 6-7: MS; 8-9: S

Supplementary Table S14: Primer sequence of markers used for foreground selection

Gene	Marker name	Forward / Reverse	Primer sequence	Chromosome	Linkage distance	Ref
Pi54	*Pi54* MAS	F	5’-CAATCTCCAAAGTTTTCAGG-3’	11	Gene based	42
		R	5’-GCTTCAATCACTGCTAGACC-3’			
Pi1	RM224	F	5’-ATCGATCGATCTTCACGAGG-3’	11	0.0 cM	43
		R	5’-TGCTATAAAAGGCATTCGGG-3’			
Pita	YL155/87	F	5’-AGCAGGTTATAAGCTAGGCC-3’	12	Gene based	44
		R	5’-CTACCAACAAGTTCATCAAA-3’			
	YL155/83	F	5’-CAACAATTTAATCATACACG-3’			
		R	5’-ATGACACCCTGCGATGCAA-3’			

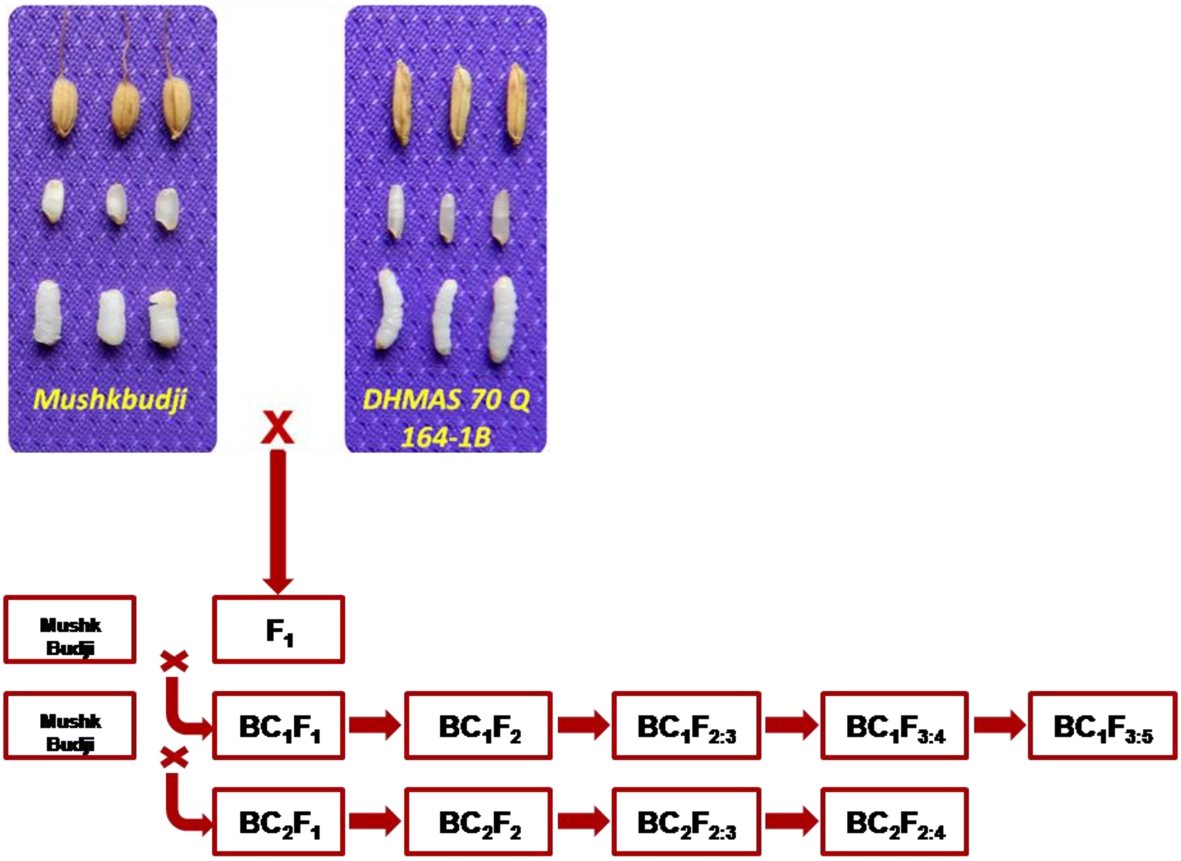

Supplementary Fig. S1: Marker-assisted backcross breeding scheme adopted for transfer of genes *Pi54+Pi1+Pita*. Seventeen BC_1F_1 plants were generated. Marker-assisted foreground analysis (FG) helped to identify plants carrying following gene combinations: *Pi54+Pita+Pi1* (SKAU-485-27)=1; *Pi54+Pi1*= 6; *Pi54*= 3; *Pi1*= 6; Recessive=1; 234 BC_2F_1 plants were produced; 45 of which came from backcrossing of single three-gene heterozygote namely, SKAU-485-27; FG helped to select the plants carrying: *Pi54+Pi1+Pita*=2; *Pi54+Pi1*= 13; *Pi54*= 5; *Pi1*= 5; *Pita*=10; recessive alleles= 3; and four were not harvested; 789 BC_1F_2 plants were harvested of which 86 analyzed were obtained from selfing of single three-gene heterozygote (SKAU-485-27). FG of 86 plants revealed: *Pi54+Pi1+Pita*=2; *Pi54+Pi1*= 10; *Pi54+Pita*= 1; *Pi1+Pita*= 1; 49 BC_2F_2 plants were put to FG out of plants obtained from selfing of two triple-heterozygotes. These yielded *Pi54+Pi1+Pita*=3; Pi1= 8; *Pita*= 23; 14 $BC_1F_{2:3}$ families (two from two 3-gene pyramids) comprising 1400 plants were grown of which 104 selected; 3 $BC_2F_{2:3}$ families (from three 3-gene pyramids) comprising 1500 plants were grown; 30 single plants selections (SPS) made per family and bulked; Nine $BC_1F_{3:4}$ SPS grown and harvested in bulk; Three $BC_2F_{2:3}$ pyramided lines grown across five locations; Nine $BC_1F_{3:5}$ pyramided lines grown across five locations.

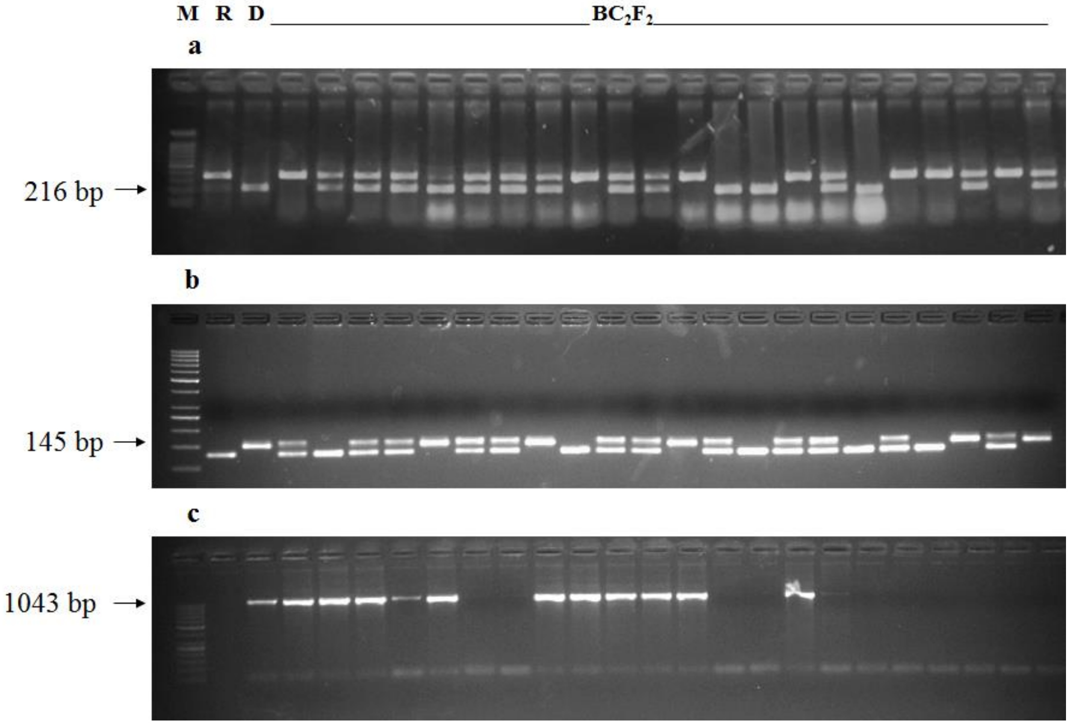

Supplementary Fig. S2: Foreground selection in BC_2F_2 for the genes *Pi54, Pi1* and *Pita*
a: InDel marker Pi54 MAS; b: SSR marker RM224; c: SNP based dominant marker YL155/87; Lanes M: 50bp size standard (Fermentas, Lithuania, U.S.); R: Mushk Budji; D: DHMAS 70Q 164-1b; Lanes 1-22: BC_2F_2 single plant selections from three-gene BC_2F_1. Lane 7: Plant# SKUA-485-27-4 homozygous for three genes (*Pi54+Pi1+Pita*). (Refer Supplementary Fig. S12-14)

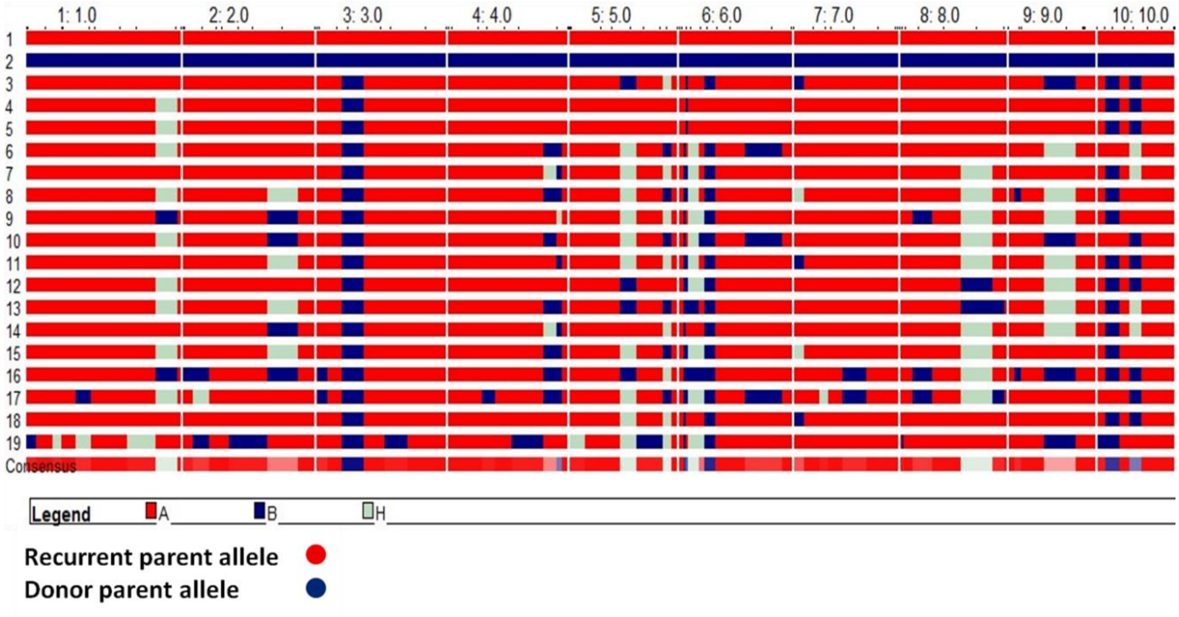

Supplementary Fig. S3: Graphical genotype of marker-assisted background selection (on non-carrier chromosomes) of pyramided lines carrying blast resistance genes *Pi54, Pi1 and Pita*. Bar1: Mushk Budji; Bar 2: DHMAS 70Q 164-1b; Bar 3-19: Backcross derived lines.

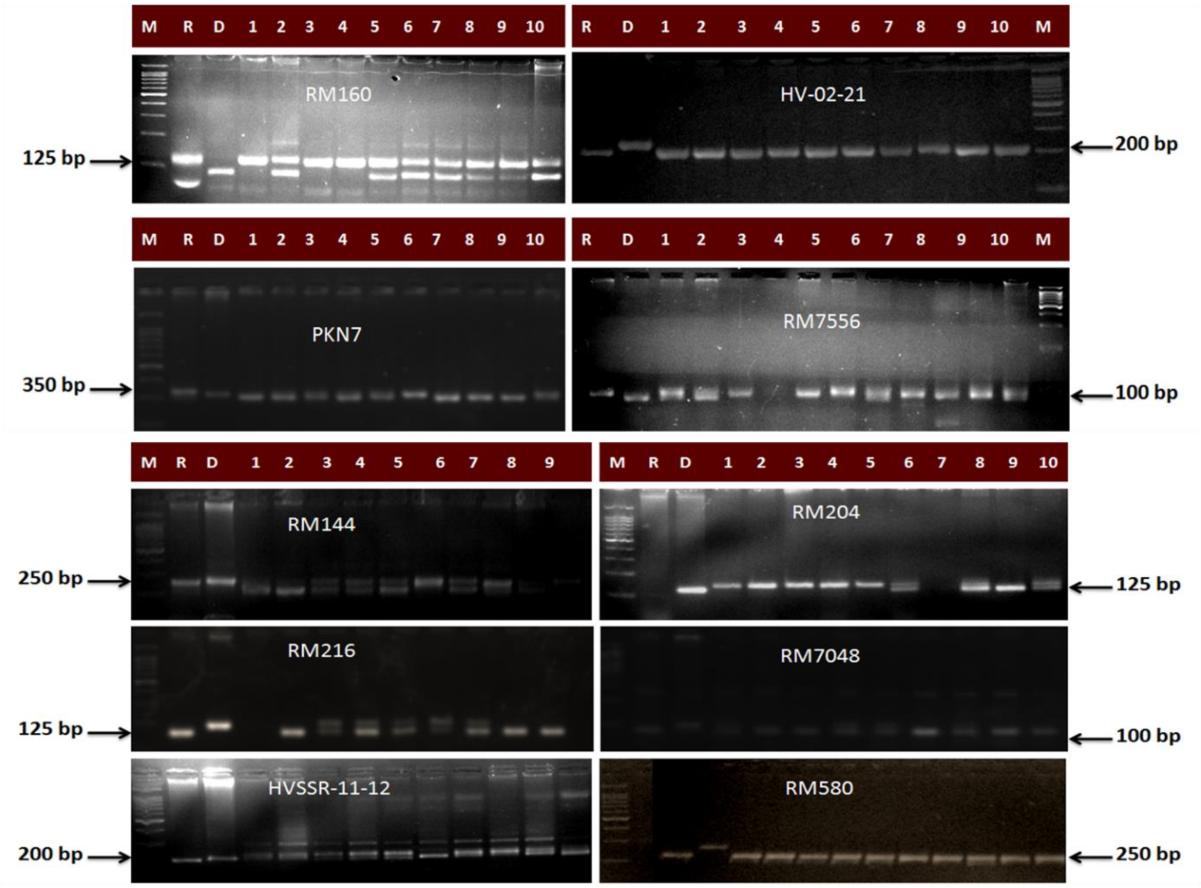

Supplementary Fig. S4: Gel image depicting recurrent parent genome recovery of pyramided lines carrying blast resistance genes *Pi54, Pi1 and Pita*. R: Mushk Budji; D: DHMAS 70Q 164-1b; Lane 1-10: Backcross derived lines; M: 50 bp size standard (Fermentas, Luthuania, U S).

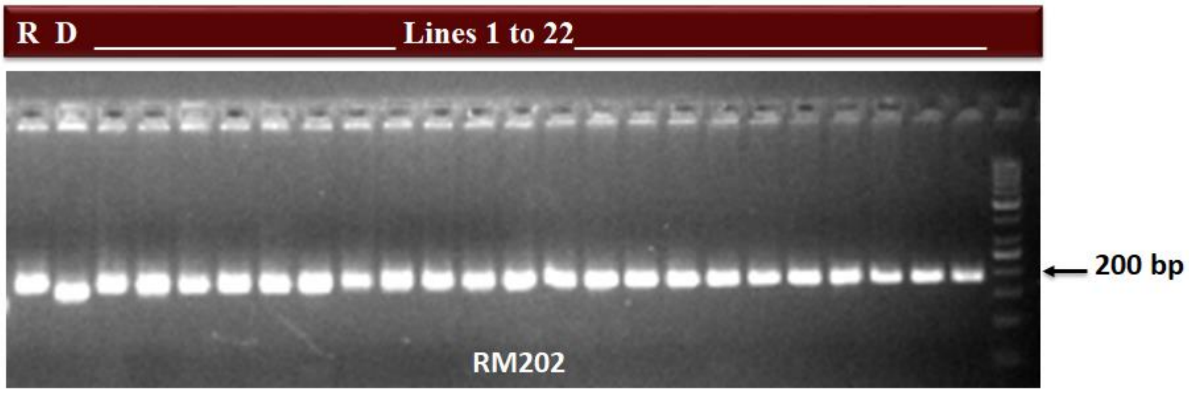

Supplementary Fig. S5: Gel image depicting recurrent parent genome recovery of pyramided lines. R: Mushk Budji; D: DHMAS 70Q 164-1b; Lane 1-22: Backcross derived lines; M: 50 bp size standard (Fermentas, Luthuania, U S)

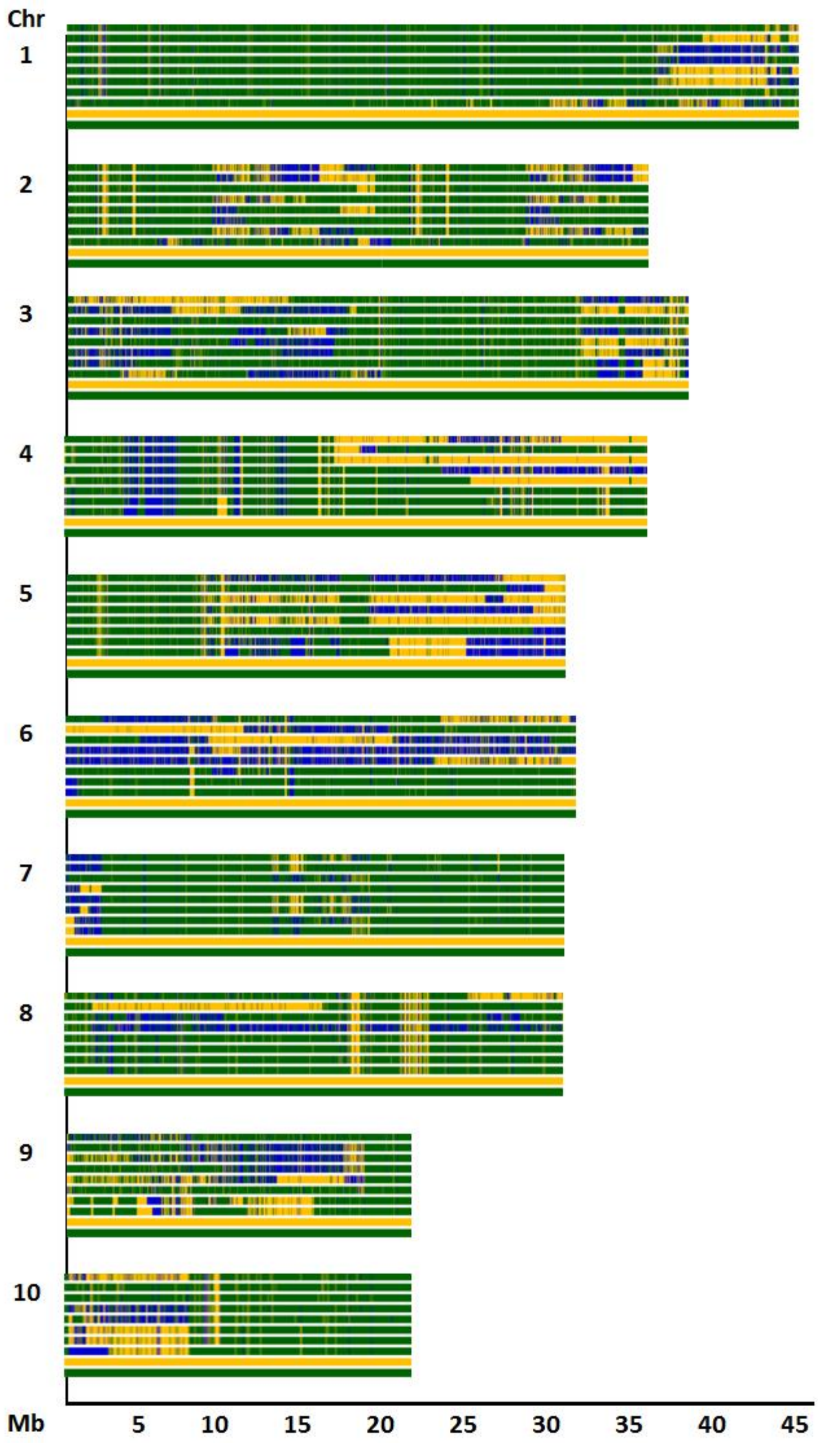

Supplementary Fig. S6: RPG recovery of pyramided lines based on 'OsSNPnks' 50K Axiom® 2.0 SNP array for non-carrier chromosomes. Bar number 1-10: Mushk Budji; DHMAS 70Q 164-1b; SKUA-485-27-4-38-4; SKUA-485-27-4-40-6; SKUA-485-27-3-7-5;SKUA-485-27-13-1-3; SKUA-485-27-20-6-4; SKUA-485-27-47-4-1; SKUA-485-27-77-6-2; SKUA-485-27-86-10-4; Yellow: Recurrent parent allele; Green: Donor allele; Blue: Heterozygous allele; x-axis represents the Mb position of respective chromosomes, 1 to 10.

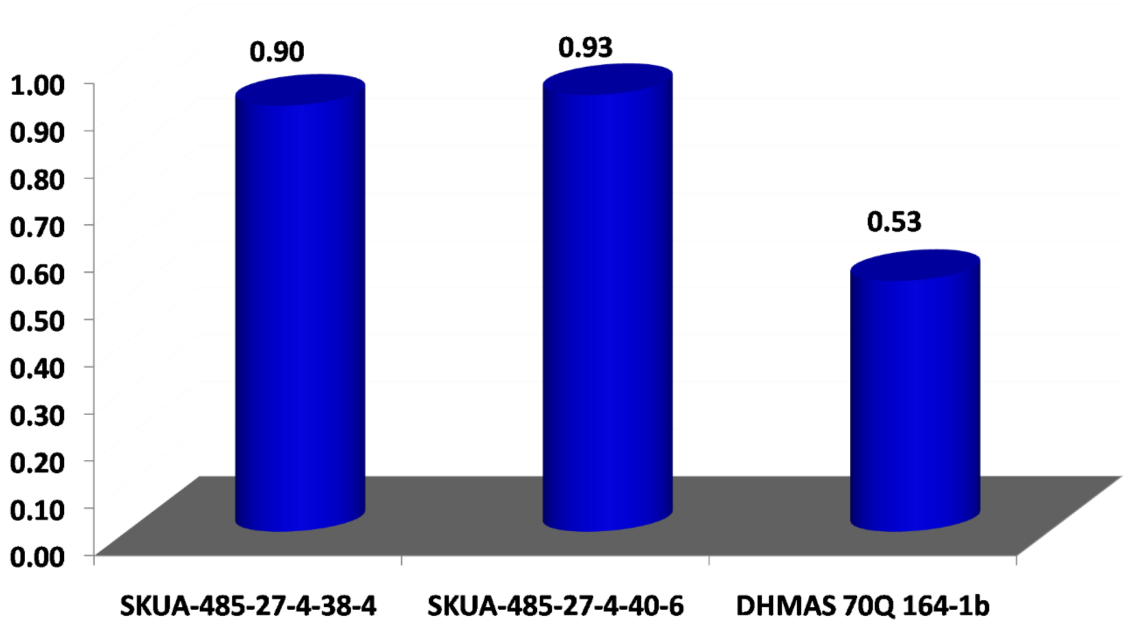

Supplementary Fig. S7: Protein match score of pyramided lines and donor with respect to recurrent parent in 2D-Gel electrophoresis. SKUA-485-27-4-38-4 andSKUA-485-27-4-40-6 share 90 and 93% of protein identity with RP *Mushk Budji* as compared to donor DHMAS 70Q 164-1b that shows only 53% similarity.

SKUA-485-27-4-40-6 SKUA-485-27-4-38-4 DHMAS 70Q 164-1b Mushk Budji

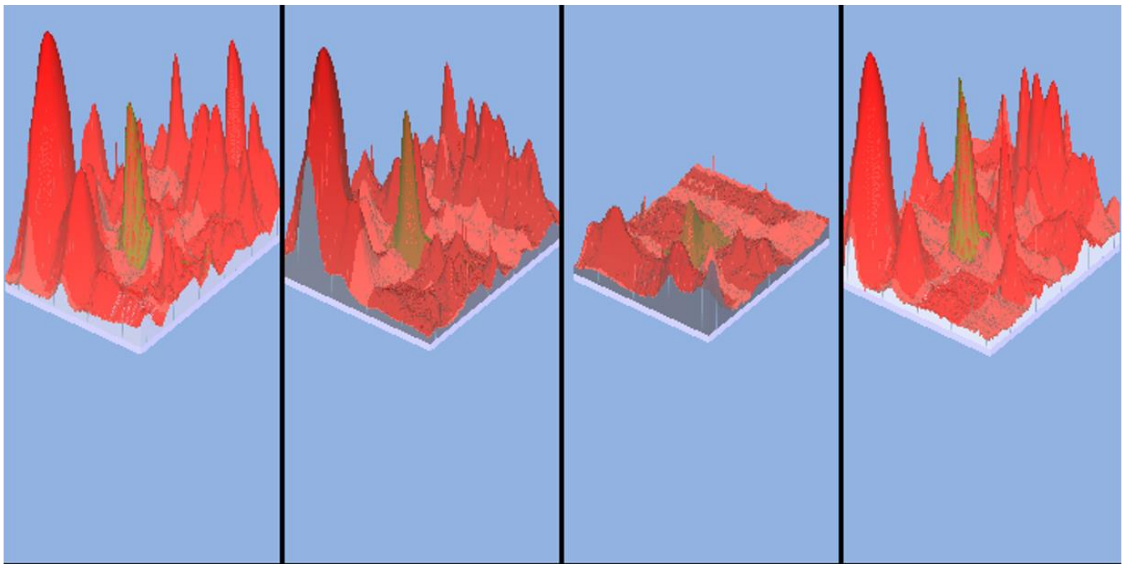

Protein	Match ID	Fold	Match Count	Mushk Budji	SKUA-485-27-4-38-4	SKUA-485-27-4-40-6	DHMAS 70Q 164-1b	ANOVA
Alpha-amylase	33	3.60399	4	0.75007	0.70812	0.74073	0.41193	0.00467

Supplementary Fig. S8: Fold change in protein expression of pyramided lines and RP in comparison to donor line for Alpha-amylase

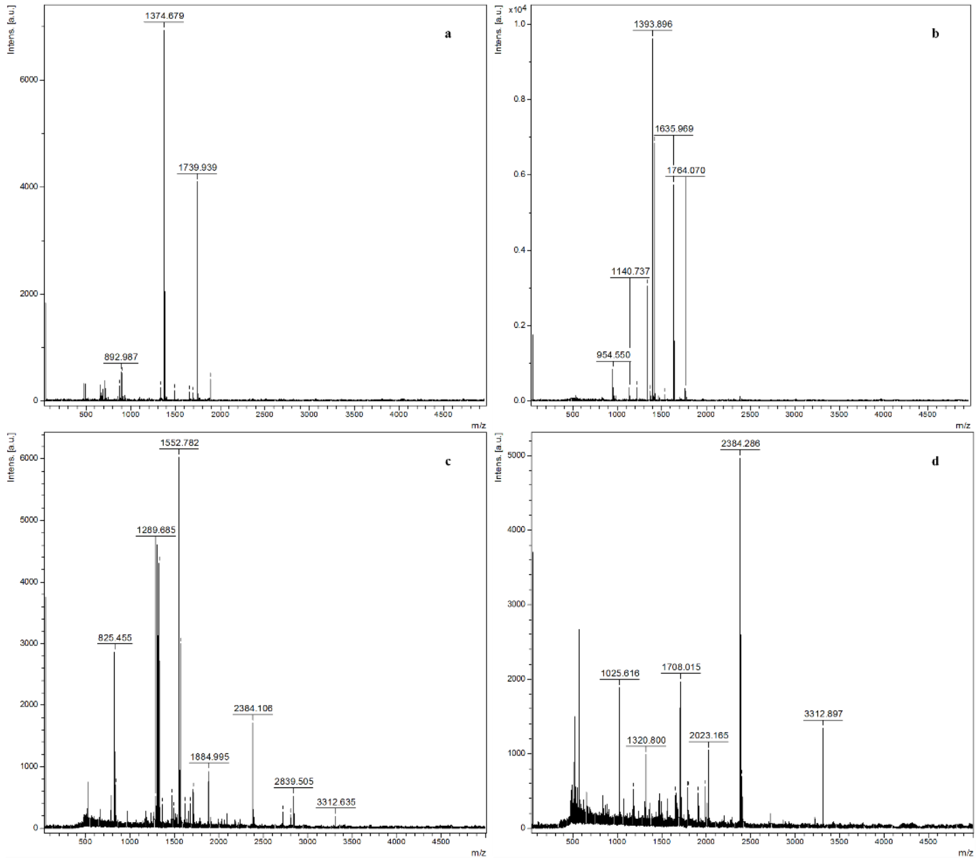

Supplementary Fig. S9: MALDI-TOF analysis of spots in recipient parent *Mushk Budji* / donor DHMAS 70Q 164-1b. a: Alpha-amylase; b: Triosephosphate isomerase; c: 19 kDa globulin; d: S-(+)-linalool synthase.

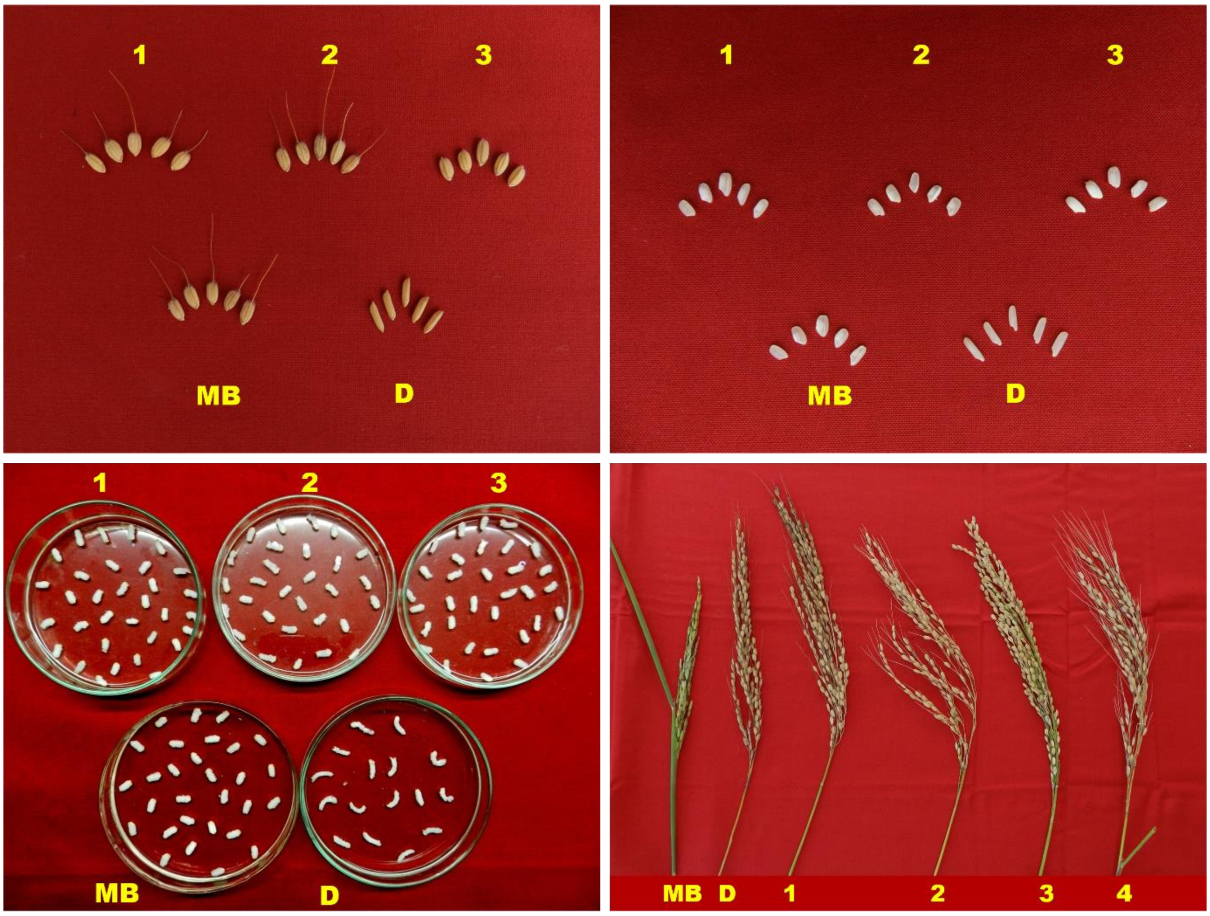

Supplementary Fig. S10: Recovery in grain type, kernel features, cooking quality and panicle attribute of pyramided lines. MB: Mushk Budji; D: DHMAS 70Q 164-1b; 1: SKUA-485-27-4-38-4; 2: SKUA-485-27-4-40-6; 3: SKUA-485-27-20-6-4; 4: SKUA-485-27-3-7-5.

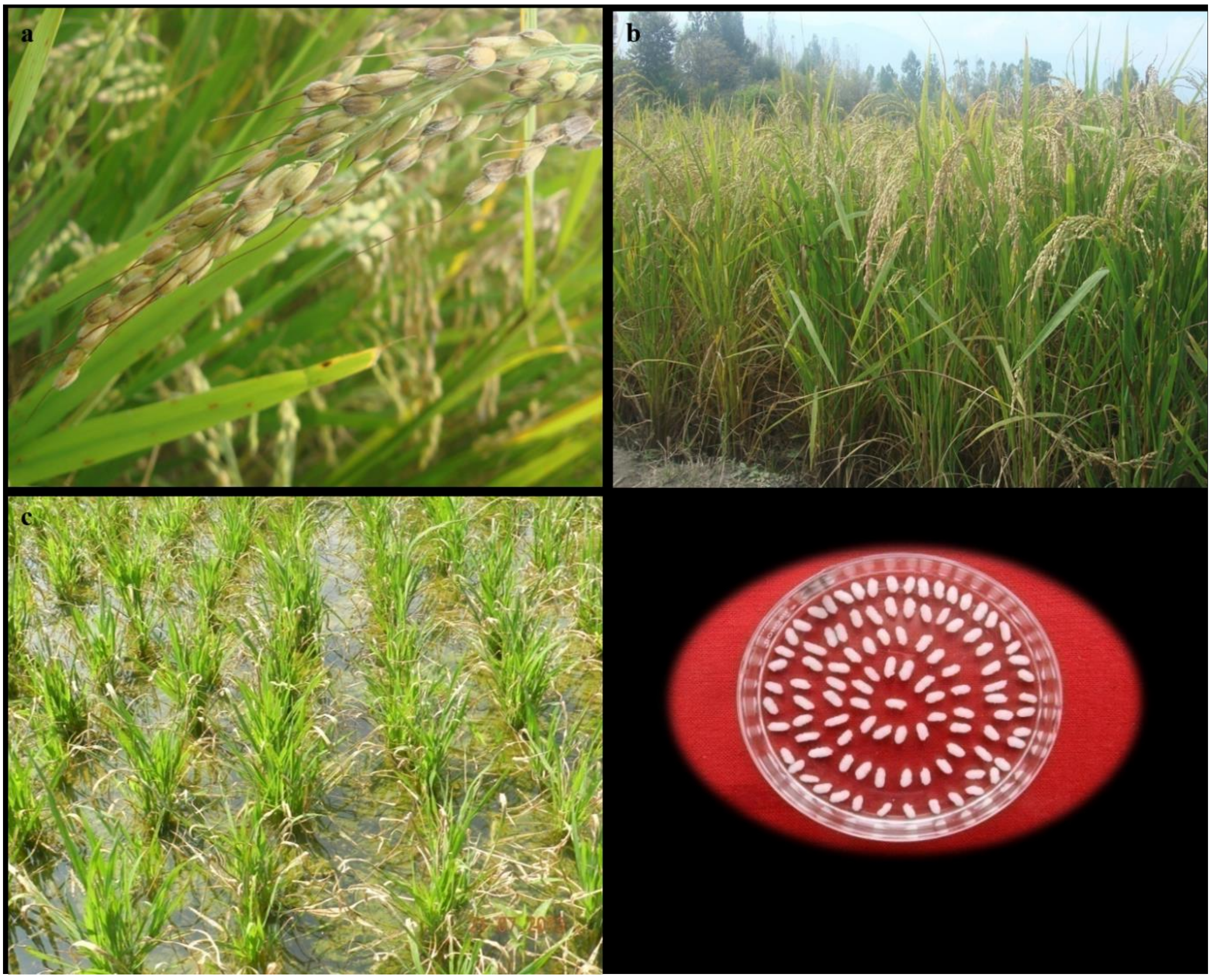

Supplementary Fig. S11: Morphological features of recurrent parent Mushk Budji. a: Panicle and grain type; b: Plant architecture; c: Mushk Budji at early seedling stage showing heavy blast incidence on leaves; d: Cooked rice.

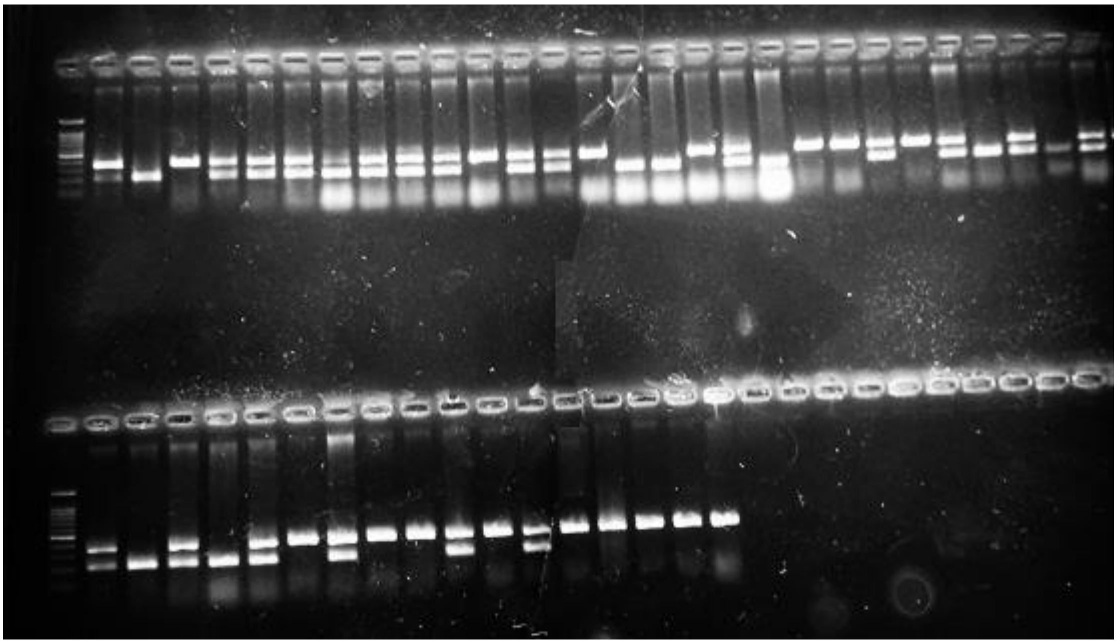

Supplementary Fig. S12: Foreground selection in BC_2F_2 for blast resistance gene *Pi54* using InDel marker Pi54 MAS.

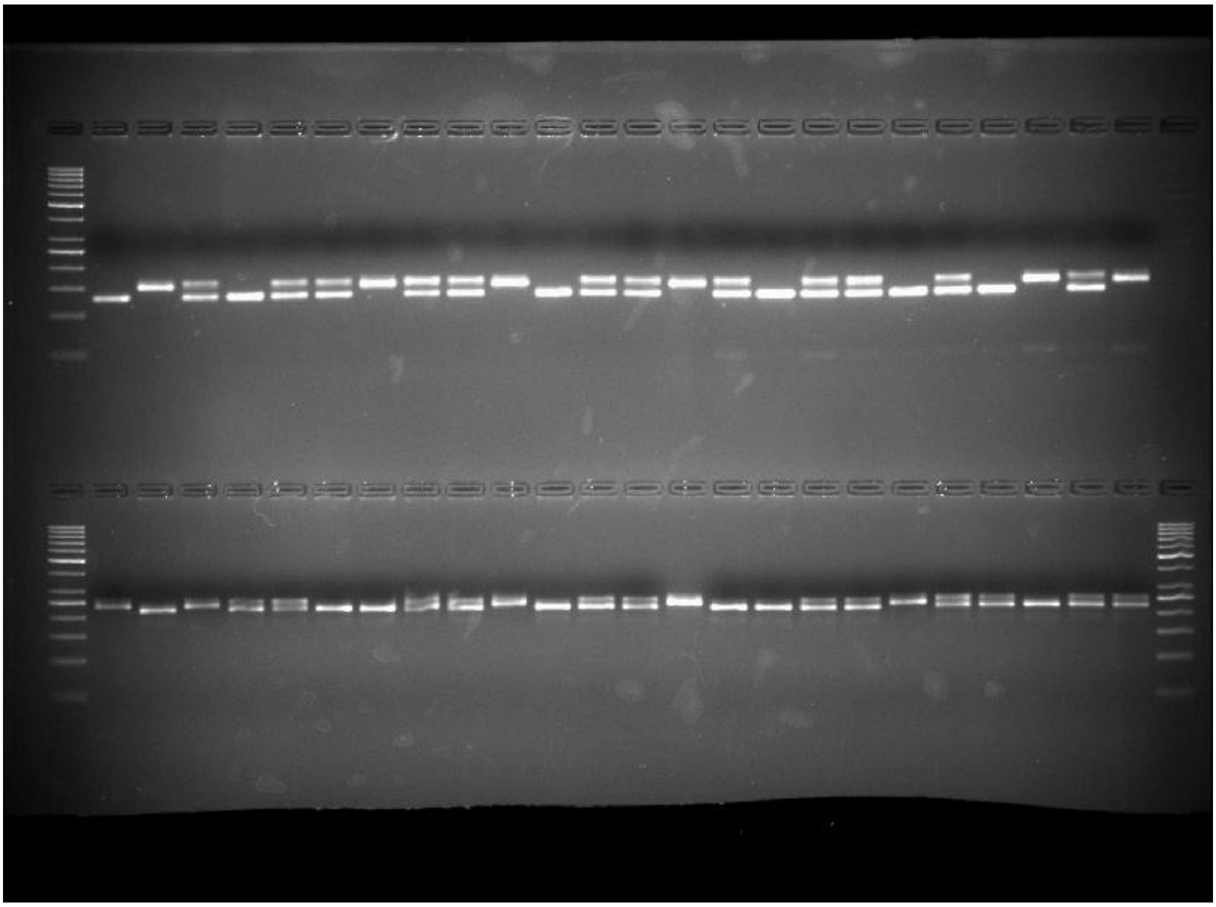

Supplementary Fig. S13: Foreground selection in BC_2F_2 for blast resistance gene *Pi1* (above panel) using gene linked marker RM224.

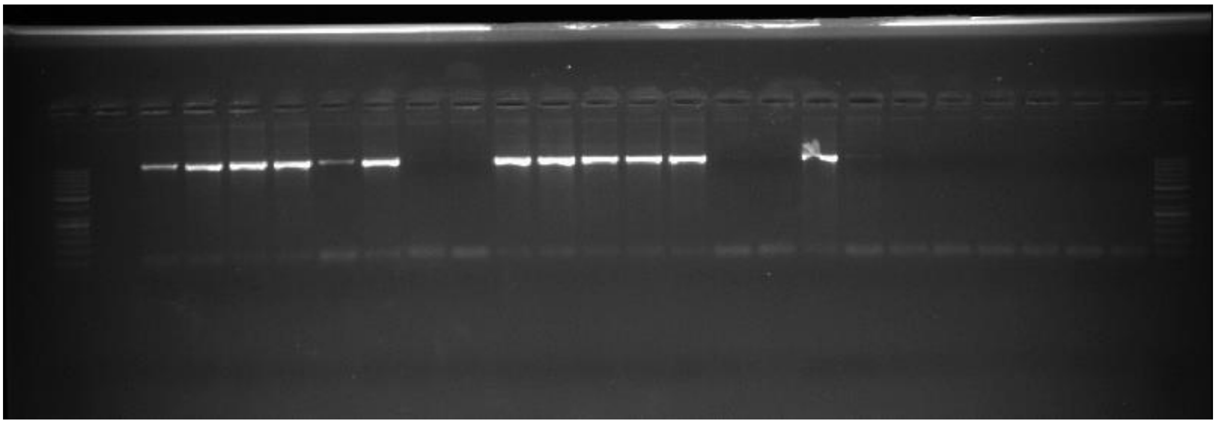

Supplementary Fig. S14: Foreground selection in BC2F2 for blast resistance gene Pita using SNP based dominant marker YL155/87.

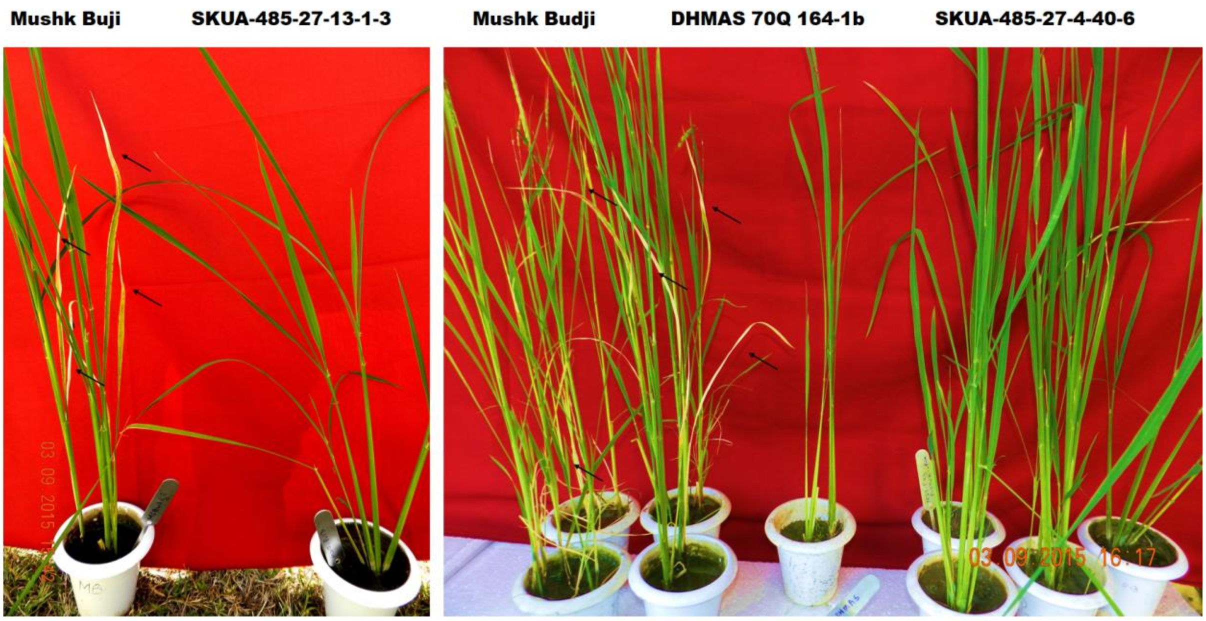

Supplementary Fig. S15: The reaction of pyramided lines to isolate Mo-ei-MBI-2. Arrow heads point at blast lesions.

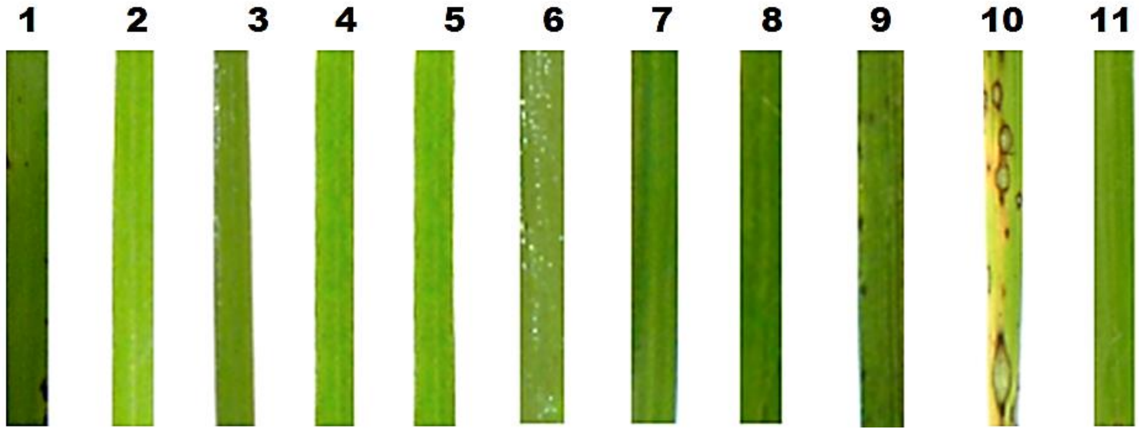

Supplementary Fig. S16: The reaction of pyramided lines to isolate Mo-nwi-kash-32. Sequence of pyramided lines screened under controlled conditions: 1: SKUA-485-27-50-5-5 (1); 2: SKUA-485-27-20-6-4 (0); 3: SKUA-485-27-13-1-3 (0); 4: SKUA-485-27-86-10-4 (0); 5: SKUA-485-27-77-6-2 (0); 6: SKUA-485-27-47-4-1 (0); 7: SKUA-485-27-3-7-5 (0); 8: SKUA-485-27-4-38-4 (0); 9: SKUA-485-27-4-40-6 (1); 10: Mushk Budji (5); 11: DHMAS 70Q 164-1b (0). The values in parenthesis indicate blast score as defined in Table 3.

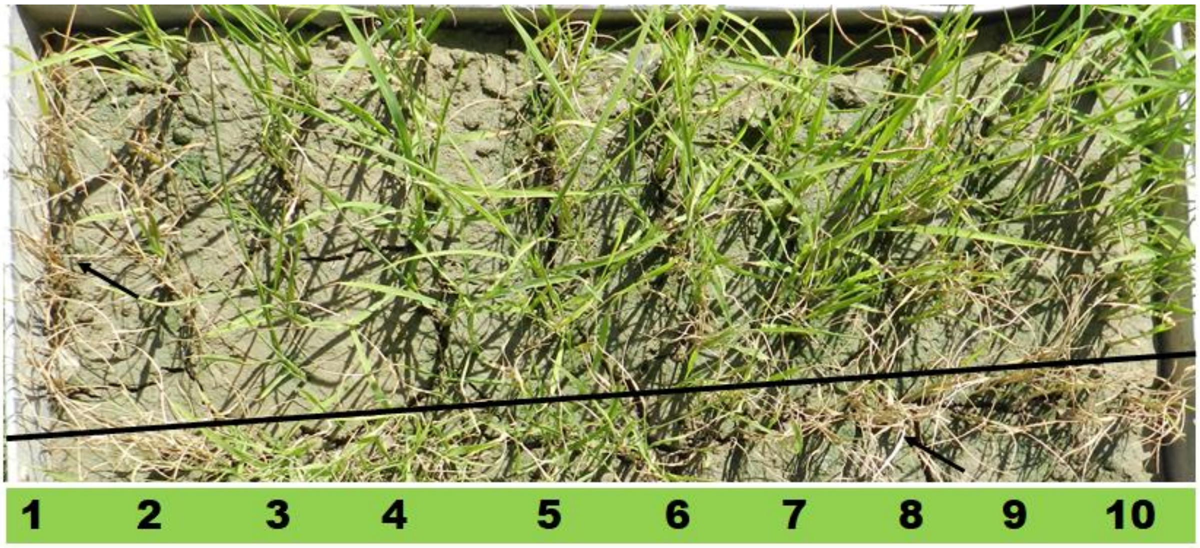

Supplementary Fig. S17: The reaction of pyramided lines to isolate SKUA-Mo-3. Sequence of pyramided lines screened under controlled conditions: 1: Mushk Budji; 2: SKUA-485-27-50-5-5; 3: SKUA-485-27-20-6-4; 4: SKUA-485-27-13-1-3; 5: SKUA-485-27-86-10-4; 6: SKUA-485-27-77-6-2; 7: SKUA-485-27-47-4-1; 8: SKUA-485-27-3-7-5; 9: SKUA-485-27-4-38-4; 10: SKUA-485-27-4-40-6. Mushk Budji was also planted as a border row as demarcated by a line; Arrow heads indicate Mushk Budji line showing susceptible reaction.

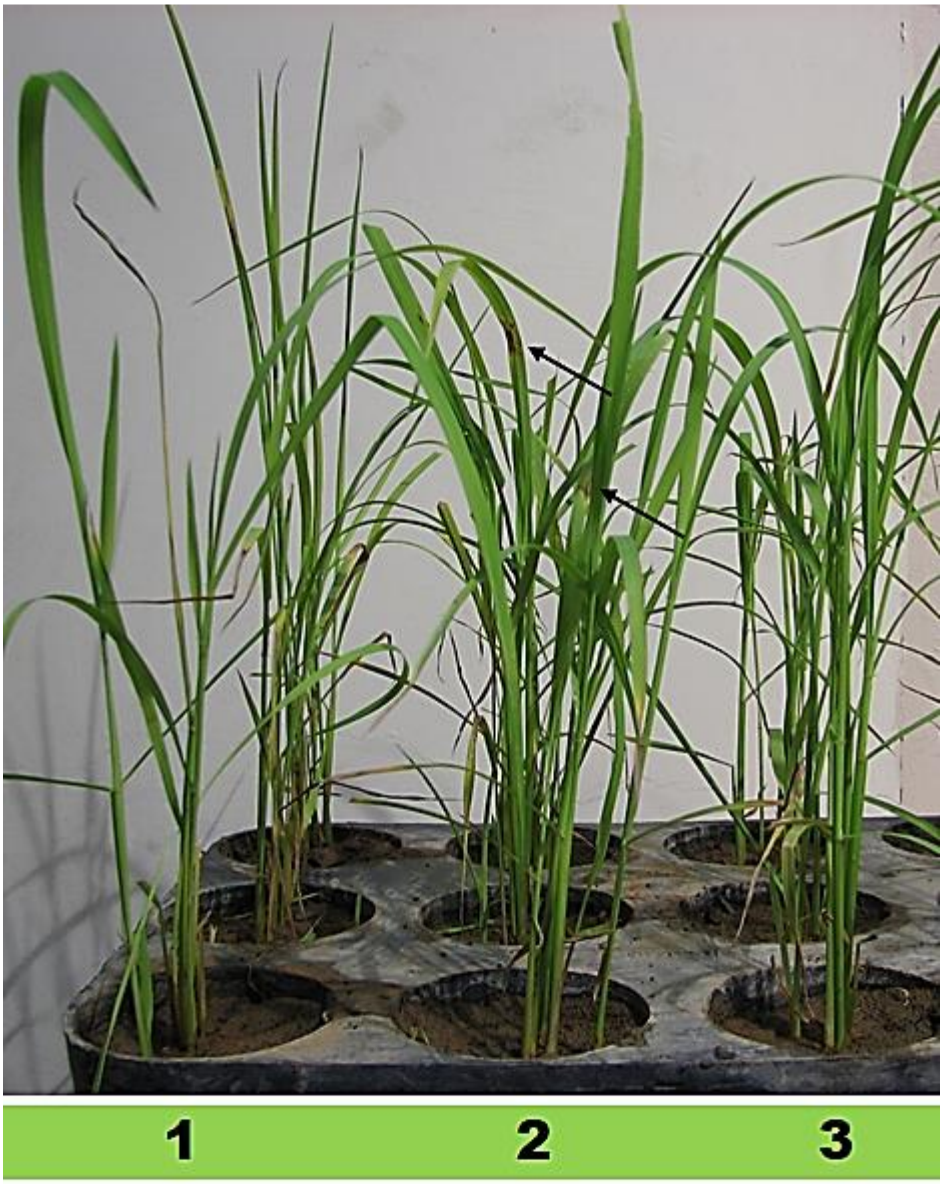

Supplementary Fig. S18: The reaction of pyramided lines to isolate SKUA-Mo-9. Sequence of pyramided lines screened under controlled conditions: 1: SKUA-485-27-50-5-5; 2: Mushk Budji; 3: SKUA-485-27-4-38-4. Arrow marks point at blast lesions.
